# Mild hydrolysis of chemically stable valerolactams by a biocatalytic ATP-dependent system fueled by metaphosphate†

**DOI:** 10.1039/d3gc04434c

**Published:** 2023-12-07

**Authors:** Sebastian Roth, Somayyeh Gandomkar, Federico Rossi, Mélanie Hall

**Affiliations:** a Institute of Chemistry, University of Graz Heinrichstrasse 28 8010 Graz Austria melanie.hall@uni-graz.at; b BioHealth, University of Graz Heinrichstrasse 28 8010 Graz Austria

## Abstract

Medium-sized 5- and 6-membered ring lactams are molecules with remarkable stability, in contrast to smaller β-lactams. As monomers, they grant access to nylon-4 and nylon-5, which are alternative polyamides to widespread caprolactam-based nylon-6. Chemical hydrolysis of monocyclic γ- and δ-lactams to the corresponding amino acids requires harsh reaction conditions and up to now, no mild (enzymatic) protocol has been reported. Herein, the biocatalytic potential of a pair of heterologously expressed bacterial ATP-dependent oxoprolinases – OplA and OplB – was exploited. Strong activity in the presence of excess of ATP was monitored on δ-valerolactam and derivatives thereof, while trace activity was detected on γ-butyrolactam. An ATP recycling system based on cheap Graham's salt (sodium metaphosphate) and a polyphosphate kinase allowed the use of catalytic amounts of ATP, leading to up to full conversion of 10 mM δ-valerolactam at 30 °C in aqueous medium. Further improvements were obtained by co-expressing OplA and OplB using the pETDuet1 vector, a strategy which enhanced the soluble expression yield and the protein stability. Finally, a range of phosphodonors was investigated in place of ATP. With acetyl phosphate and carbamoyl phosphate, turnover numbers up to 176 were reached, providing hints on a possible mechanism, which was studied by ^31^P-NMR.

## Abstract 2

The biocatalytic hydrolysis of valerolactams was achieved with ATP-dependent oxoprolinases OplAB, coupled to an *in situ* ATP regeneration using metaphosphate.

## Graphical abstract

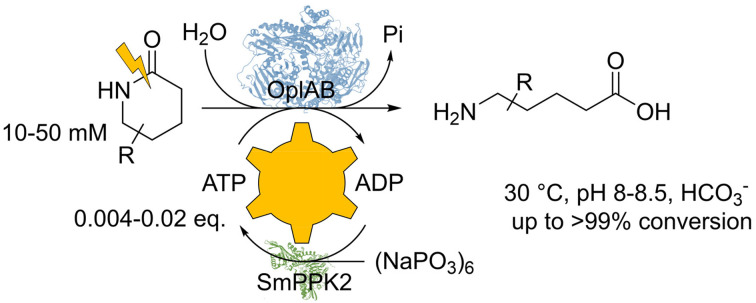

## Introduction

Polyamides are synthetic polymers obtained by the repetition of units linked by amide bonds and broadly described as nylon. Based on the repeating units, different types of nylon are obtained, such as the emblematic nylon-6, predominantly used in the textile and plastic industries and produced through the ring-opening polymerization of ε-caprolactam.^1,2^ Nylon-5 is obtained in a similar manner from δ-valerolactam and displays ferroelectric properties and high melting point above 250 °C.^3^ Nylon-5,6 is obtained by the copolymerization of ε-caprolactam and δ-valerolactam and exhibits superior properties as fabric in terms of elastic recovery and moisture-absorbance properties.^4,5^ The manufacturing of these polymers implies problematic consequences for the environment through the release of unreacted monomers§§Hazard pictograms are reported for both γ-butyrolactam (GHS07 and GHS08) and δ-valerolactam (GHS07), see ESI† for details. into the wastewater, which thus needs to be treated before release.^6^ While biological treatments of these wastewaters are still rare, microorganisms able to degrade ε-caprolactam have been identified.^6^ Such approach is more challenging in the context of medium-sized lactams, especially γ-butyrolactam and δ-valerolactam, because of their exceptional chemical stability connected to the low ring strain. The increased resonance stabilization of the amide bond and the higher partial C–N double bond character in these lactams are responsible for the resistance of the carbonyl group to hydrolysis, and in general, to nucleophilic attack.^7,8^ The hydrolysis of such molecules requires in fact harsh reaction conditions, such as boiling under reflux in strongly acidic solutions for extended times.

**Fig. 1:**
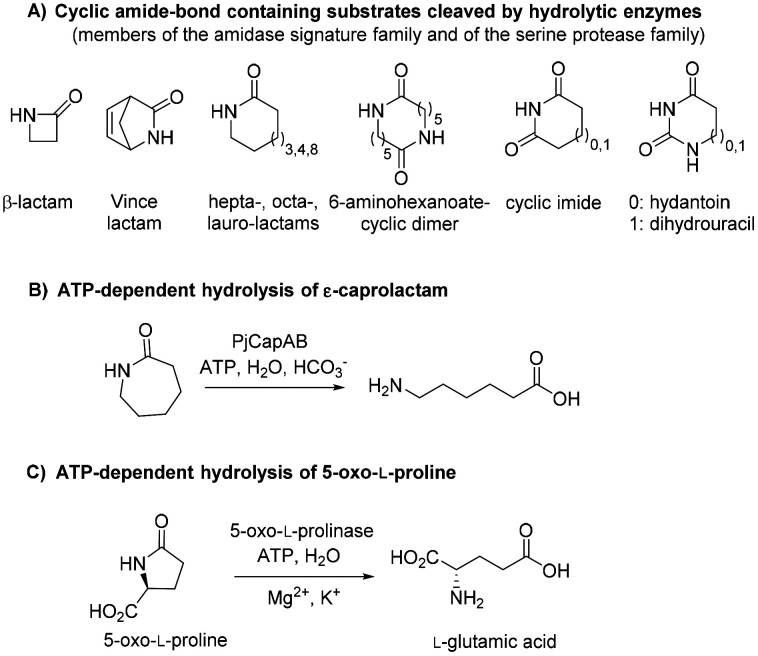
State-of-the art in the biohydrolysis of lactams and lactam-like compounds: (A) cyclic amide-bond containing substrates cleaved by hydrolytic enzymes;^7,15,16^ (B) ATP-dependent hydrolysis of ε-caprolactam by PjCapAB;^18,19^ (C) hydrolysis of oxoproline by ATP-dependent oxoprolinase.^20^

Biotechnological access to δ-valerolactam is currently being investigated as an approach to accessing bio-sourced plastics.^9^ However, the challenging biodegradability of this molecule may hamper its wider acceptance. To the best of our knowledge, no enzymatic approach has been identified for the mild cleavage of γ-butyrolactam and δ-valerolactam, and surprisingly, a variety of hydrolytic enzymes were shown inactive on these simple molecules.^7,10^ Slight modifications of the ring backbone have important effects on the reactivity, and active enzymes could be identified for bicyclic structures of the type of (±)-2-azabicyclo[2.2.1]hept-5-en-3-one (*i.e. rac*-Vince lactam, Fig. 1A). These enzymes include members of the amidase signature family and members of the serine protease family, however, all were inactive on monocyclic γ- and δ-lactams.^10^ While the absence of proteins active on the amide bond of γ- and δ-lactams from the biocatalytic repertoire of cleaving enzymes is striking, related amide bond-containing monocyclic compounds of similar ring size, or further functionalized, seem less resistant to biohydrolysis.^7^ Other examples include 8-, 9- and 13-membered ring lactams,^7,11^ cyclic imide,^12^ 6-aminohexanoate-cyclic dimer,^13^ or dihydrouracil (Fig. 1A).^14^ Rich enzymatic diversity promotes hydrolysis of these electronically unrelated structures *via* distinct mechanisms. Active hydrolases relying on a serine-triggered nucleophilic attack of the substrate carbonyl group are frequent and are complemented by mostly zinc-dependent metalloproteins, such as dihydropyrimidinases, hydantoinases, or allantoinases (broadly classified as cyclic amidohydrolases^15,16^), in which direct attack of the carbonyl group by an activated water molecule is facilitated by substrate coordination to a Lewis acid-acting Zn^2+^.^17^ Finally, the reactivity of β-lactams is due to the strong ring strain of the small lactam ring.^7^

Recently, an ATP-dependent enzymatic system involved in the degradation pathway of ε-caprolactam was identified in *Pseudomonas jessenii* by proteomics analysis.^18,19^ The two subunits PjCapA and PjCapB function in pair and were shown to catalyze the hydrolysis of ε-caprolactam (Fig. 1B). On a sequence level, the two enzymes are closely related to oxoprolinases OplA and OplB, which are responsible for the endergonic ring opening of l-pyroglutamic acid (5-oxo-l-proline) to form l-glutamic acid with concomitant hydrolysis of ATP (Fig. 1C).^20^ While bacterial homologues are made of two separate fragments encoded by two distinct genes,^20^ eukaryotic oxoprolinases are large one-fragment proteins (*e.g.*, rat Oplah MW: ∼137 kDa).^18^ These enzymes are also related to ATP-dependent carboxylases, such as acetone and acetophenone carboxylases.^19^ Hydrolysis of ε-caprolactam to the corresponding ω-amino acid 6-aminocaproic acid by PjCapAB was demonstrated on 2 mM substrate in the presence of 1 equivalent of ATP, leading to the formation of 1.4 mM of product. Since the presence of bicarbonate was found necessary for the formation of the product, and highest velocity was obtained in the presence of a large excess of bicarbonate (50 mM), bicarbonate was postulated to be involved in the reaction mechanism through the formation of a carboxyphosphate intermediate from ATP.^19^ This hypothesis was derived from analogy with related enzymes acetone and acetophenone carboxylases. ATP-dependent phosphorylation of bicarbonate is indeed a vital process in a number of biological processes, through which carboxyphosphate can transfer the carboxy group to a variety of acceptors, including acetone and acetophenone,^21,22^ or ammonia.^23^

**Fig. 2:**
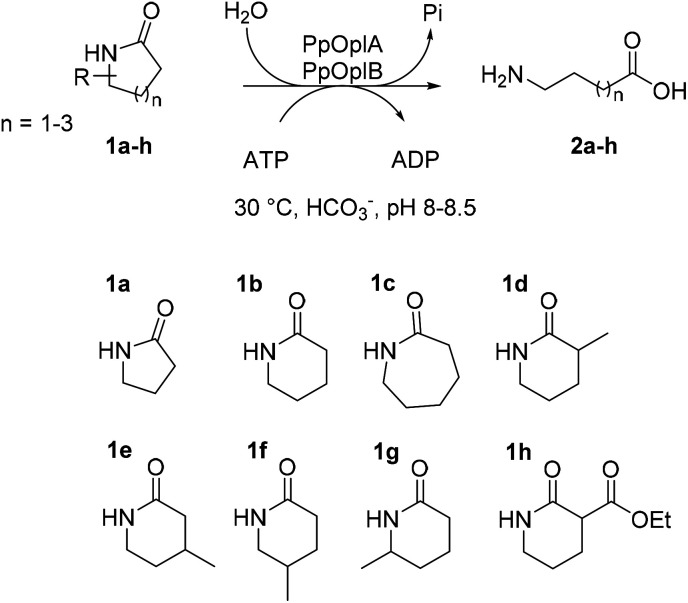
Enzymatic hydrolysis of medium-sized lactams to the corresponding ω-amino acids by the pair of ATP-dependent oxoprolinases OplA and OplB from *Pseudomonas putida* KT2440 and overview of investigated lactams 1a–h.

OplAB from *Pseudomonas putida* KT2440 was recently shown to be involved in δ-valerolactam catabolism in the context of whole cells utilizing this molecule as sole carbon source, however no activity in the hydrolysis of δ-valerolactam toward the formation of 5-aminovaleric acid could be detected with the purified proteins *in vitro*. Involvement of these enzymes in the degradation of ε-caprolactam was also demonstrated in the whole cells.^24^ With the goal to establish a generally applicable biocatalytic platform for the ring opening of γ- and δ-lactams under mild reaction conditions, and given the sequence identity between PpOplA and PjCapA (80%) and between PpOplB and PjCapB (86%), we decided to explore the catalytic activity of the OplAB system from *Pseudomonas putida* KT2440 in the ring cleavage of a range of chemically stable lactams toward the corresponding ω-amino acids (Fig. 2). Our strategy relied on the heterologous expression of both enzymes in *E. coli* and their investigation in hydrolytic reactions *in vitro*.

## Results and discussion

### Individual access to PpOplA and PpOplB and activity assays

The synthetic genes coding for PpOplA and PpOlpB, both flanked with the restriction sites *Nde*I at the N-terminus and *Hind*III at the C-terminus and codon optimized for expression in *E. coli*, were ordered and cloned in the vector pET28a(+). Given our interest to study the effect of both proteins separately in view of getting insights on the role of each in the mechanism, we decided to attempt their over-expression individually. The heterologous over-expression of both proteins carrying a N-terminal His-tag in *E. coli* BL21 (DE3) in lysogeny broth under isopropyl β-d-1-thiogalactopyranoside (IPTG) induction at 30 °C resulted in poor soluble expression, as was the case in trials at lower temperatures (20 °C, data not shown), despite good overall over-expression (see ESI, Fig. S1†). The expression was thus performed under autoinduction conditions. The presence of ammonium bicarbonate in the lysis buffer during cell lysis by sonication was crucial for the release of soluble protein (see ESI, Fig. S2 and S3†), especially in the case of PpOplA, while in general PpOplB showed low soluble expression or low release as soluble protein. Due to the very low amounts of PpOplB obtained after purification by immobilized metal affinity chromatography (IMAC, see ESI, Fig. S4†), biotransformations were first performed by combining both cell-free extracts (CFEs) obtained by collecting both supernatants upon cell lysis by sonication.

The first tests were run on 2 mM δ-valerolactam (1b) in aqueous ammonium bicarbonate buffer (50 mM, pH 8.5) at 30 °C for 16 h in the presence of 1 eq. ATP and equal amounts of total protein in CFEs of PpOplA and PpOplB. The formation of 5-aminovaleric acid (2b) was monitored by gas chromatography after derivatization with ethyl chloroformate (see ESI, Fig. S8–S16†), and reached 1.4 mM (70% conversion), indicating strong activity of the pair PpOplA/PpOplB in the hydrolysis of 1b (Table 1, entry 2). Full conversion could be obtained in the presence of excess of ATP (2.5 eq., Table 1, entry 3). Under these conditions, no product formation could be detected with γ-butyrolactam (1a). The use of a large excess of ATP was found detrimental to the reaction with 1b as conversion dropped to 35% with 12.5 mM of ATP. Up to 80% conversion could be reached on 5 mM 1b with 7.5 mM ATP (Table 1, entry 6). Under the tested conditions, no conversion was observed on 10 mM 1b. Conversions on ε-caprolactam (1c) remained low at both 2 mM and 5 mM substrate concentration (max. 25%, Table 1, entries 9 and 10), highlighting the preference of the pair PpOplA/PpOplB for the 6-membered ring lactam. Different control reactions in the absence of ATP, PpOplA, PpOplB, or both PpOplA and PpOplB, were performed in parallel; no amino acid could be detected in any of these cases. No product could be detected when the reaction was run in potassium phosphate buffer (50 mM, pH 7.5) in absence of ammonium bicarbonate, confirming past observations with homologous PjCapAB that bicarbonate is crucial for activity.^19^ Noteworthy is that activity was strongly diminished upon freezing and thawing of the enzyme preparations, therefore CFEs were prepared fresh before each round of biotransformation. A mixture of lyophilized whole cells separately expressing PpOplA and PpOplB was found active in the hydrolysis of 2 mM 1b with 5 mM ATP, however lower conversions were achieved (max. 50% conversion, data not shown). The limitation in using a mixture of whole cells is likely due to the necessary transfer from the first intermediate obtained with PpOplA to the second biocatalyst preparation, which requires exchange from one cell to another. The use of purified proteins was impaired due to the low amounts of PpOplB retrieved (see ESI, Fig. S4†) and first attempts did not lead to any conversion (data not shown). Early studies already indicated the tendency of the purified fragment B from *Pseudomonas putida* to aggregate,^25^ affecting activity. Given the negative impact of high concentrations of ATP on conversion (see Table 1), a stepwise addition of ATP was implemented. Addition of three batches of 5 mM ATP over 2 h led to a great increase in the formation of 2b (up to >99% conversion from 6 mM 1b, and 6.9 mM 2b obtained from 8 mM 1b, Table 2, entries 1 and 2). On 1c, the effects remained modest (up to 1.4 mM 2c, Table 2, entries 4–6), confirming the preference of the PpOplAB system for the valerolactam ring under these reaction conditions.

**Table 1:** Hydrolysis of 1a–c to the corresponding amino acids 2a–c by CFEs of PpOplA and PpOplB expressed individually in *E. coli*^a^

Entry	Substrate	[1a–c] (mM)	[ATP] (mM) (eq.)	[2a–c] formed (mM)	Conv. to 2a–c (%)
1	1a	2	5 (2.5)	n.d.	n.a.
2	1b	2	2 (1)	1.4	70
3	1b	2	5 (2.5)	∼2	>99
4	1b	2	12.5 (6.25)	0.7	35
5	1b	5	5 (1)	2.1	42
6	1b	5	7.5 (1.5)	4.0	80
7	1b	5	10 (2)	3.1	62
8	1b	10	25 (2.5)	n.d.	n.a.
9	1c	2	5 (2.5)	0.5	25
10	1c	5	10 (2)	0.8	16

a Reaction conditions: ammonium bicarbonate buffer (50 mM, pH 8.5) containing MgCl_2_ (4 mM), CFEs of PpOplA and PpOplB (11.4 mg mL^−1^ each, total protein content), [ATP] as indicated, [1a–c] as indicated, incubation at 30 °C for 16 h and 140 rpm. Reactions performed in duplicates and analyzed by gas chromatography after derivatization of the amino acid and use of a calibration curve with reference materials.

**Table 2:** Stepwise addition of ATP in the hydrolysis of 1b–c by CFEs of PpOplA and PpOplB expressed individually^a^

Entry	Substrate	[1a–c] (mM)	[2a–b] formed (mM)	Conv. to 2a–b (%)
1	1b	6	∼6	>99
2	1b	8	6.9	86
3	1b	10	6.7	67
4	1c	6	1.4	24
5	1c	8	1.2	15
6	1c	10	1.2	12

a Reaction conditions: ammonium bicarbonate buffer (50 mM, pH 8.5) containing MgCl_2_ (4 mM), CFEs of PpOplA and PpOplB (11.4 mg mL^−1^ each, total protein content), ATP (15 mM final concentration, 5 mM added at 0 h, 1 h and 2 h), [1b–c] as indicated, incubation at 30 °C for 16 h at 140 rpm. Reactions performed in duplicates and analyzed by gas chromatography after derivatization of the amino acid and use of a calibration curve with reference materials.

### Co-expression of PpOplA and PpOplB in the pETDuet1 vector

**Fig. 3:**
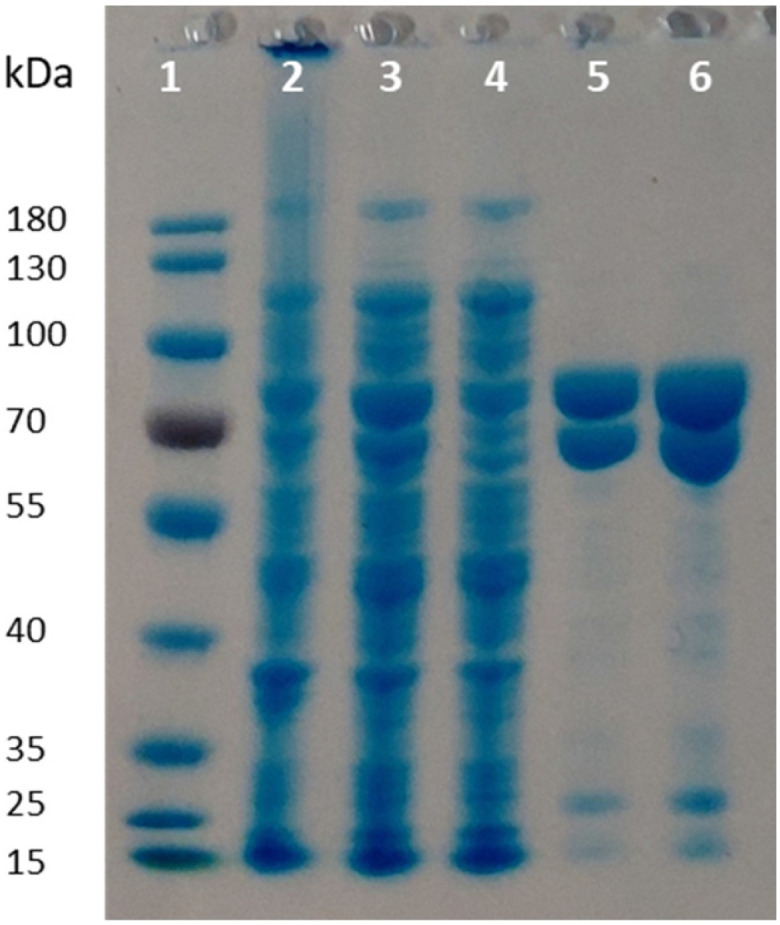
SDS-PAGE following co-expression of PpOplA and PpOplB using the pETDuet-1 vector (MW PpOplA including His-tag: ∼76 kDa, MW PpOplB: ∼63 kDa). Lane 1: PageRuler™ Prestained Protein Ladder; lane 2: pellet; lane 3: supernatant (soluble fraction); lane 4: flow through from IMAC; lanes 5 and 6: desalted purified enzyme fraction.

Given the poor stability of the enzyme preparations upon storage and the low soluble expression of PpOplB (*vide supra*), improvements in the protein expression were necessary for further investigations of the catalytic activity. We decided to co-express PpOplA and PpOplB using a co-expression vector, taking into account that the crystal structure of the homologous caprolactamase system from *Pseudomonas jessenii* revealed a heterotetrameric assembly, in which each CapB subunit interacts with a CapA subunit. We anticipated that co-expression of both subunits of the OplAB system within the same cell would favor the formation of the tetrameric assembly upon protein expression, likely impacting positively the stability of the whole assembly. In addition, such protocol grandly simplifies the preparation of the biocatalyst. The use of the pETDuet1 vector with two multiple cloning sites allowed successful co-expression of both enzymes in *E. coli* BL21 (DE3) under autoinduction in terrific broth at 24 °C. Noteworthy was the significant increase in the soluble expression of PpOplB and the comparable yields of soluble protein between PpOplA and PpOplB (Fig. 3). This method allowed the generation of a stable assembly in solution, as demonstrated by the successful co-purification of both proteins by IMAC. Since only PpOplA bears a His-tag at the N-terminus, this points out strong interactions between the two subunits and the formation of a protein complex.^26^ A 1 : 1 molar yield of the soluble assembly was confirmed by densitometric analysis of SDS-PAGE (see Fig. 3 and ESI, Table S1†). The presence of bicarbonate in the lysis buffer contributed to higher protein yields, but was found in this case not to be mandatory for good release of soluble proteins, which was also obtained in Tris and HEPES buffers for both proteins (see ESI, Fig. S5†).

The activity of co-expressed PpOplA and PpOplB (now described as PpOplAB) was investigated on a range of lactams (Fig. 2). To simplify the analysis and avoid time consuming derivatization for GC analysis, a method was developed that allowed for co-analysis of substrate and product by LC-MS, directly from the aqueous reaction mixture. Calibration curves were generated for all reference amino acids (see ESI, Fig. S17–S22†). PpOplAB was used as CFEs on 10 mM 1a–h in the presence of 12.5 mM ATP (total protein content of 8 mg mL^−1^, corresponding to ∼7 μM of PpOplAB based on densitometric analysis, see Table S1†). Strongest activity could be observed on 1b (full consumption) and 5-methylpiperidin-2-one (1f, leading to 7.9 mM product, Table 3), while conversion on 1c remained modest (2.3 mM 2c formed, Table 3, entry 3). For the first time, some product could be observed in the conversion of γ-butyrolactam (1a), corresponding to ∼4% substrate consumption (see ESI, Fig. S26†). With other methyl derivatives of δ-valerolactam, conversions were moderate (max. 2.4 mM product, Table 3) and substitution closest to the amide bond (especially 3- and 6-position) impacted the conversion negatively the most. In contrast, substitution furthest to the carbonyl had almost no impact on activity, pointing at possible steric hindrance near the reactive moiety with 1d, 1e and 1g. Finally, 3-ethoxycarbonyl-2-piperidone (1h) with a large and electron-withdrawing substituent in α-position to the carbonyl was not accepted (data not shown). All substituted lactams were tested as racemic mixtures. The strong activity on 1f already hinted at a poor enantioselectivity (79% conversion). The enantioselectivity was further looked at with compound 1e, since activity was high enough to deliver product for analysis of the enantiomeric excess (ee) while the conversion remained below 50%, which could indicate a case of kinetic resolution. A method was developed that allowed to measure the ee values for the product 2e on chiral phase HPLC after derivatization, and for the remaining substrate 1e on chiral phase GC (see ESI, Fig. S37–S39†). PpOplAB showed low (*R*)-enantioselectivity and delivered (*R*)-2e with 68% ee and remaining (*S*)-1e with 8% ee, which corresponds to an *E*-value of 6.¶¶The *E*-value was computed using the freely accessible program available at https://biocatalysis.uni-graz.at/biocatalysis-tools/enantio, with calculations based on work detailed in the literature.^32^ Collectively, the data indicate that a kinetic resolution using PpOplAB cannot deliver the amino acid with high enantiopurity. Remarkable was the tolerance of PpOplAB toward high concentrations of ATP, highlighting the advantages of the co-expression for protein stability, likely connected to a stabilizing assembly. The CFEs were also found not sensitive to freezing and thawing (Table 3, entry 8). While purification of PpOplAB was possible (see Fig. 3), the recovered proteins were poorly active on 1b under the tested conditions (max. 15% conversion to 2b, see ESI, Fig. S34†). All further reactions were therefore performed with CFEs.

**Table 3:** Hydrolysis of lactams 1a–h with co-expressed PpOplAB^a^

Entry	Substrate	[2a–h] (mM)	Conv. to 2a–h (%)
1	1a	n.d.^b^	∼4
2	1b	7.7 (±0.8)	77^c^
3	1c	2.3 (±0.1)	23
4	1d	1.3 (±0.1)	13
5	1e^d^	2.4 (±0.1)	24
6	1f	7.9 (±0.3)	79
7	1g	1.4 (±0.0)	14
8	1h	n.f.	n.a.

a Reaction conditions: 8 mg mL^−1^ PpOplAB CFE (total protein content), 10 mM 1a–h, 12.5 mM ATP, ammonium bicarbonate buffer (39 mM, pH 8.5), 8 mM MgCl_2_, 30 °C, 900 rpm, 24 h. Reactions performed in triplicates and analyzed by LC-MS (standard deviation indicated).

b n.d.: not determined, product formation not quantified. 4% substrate consumption (see ESI, Fig. S26†). n.f.: not formed, n.a.: not applicable.

c Substrate fully converted. See ESI, Fig. S26–S33.†

d *E*-Value of 6, see text and ESI.†

The influence of the bicarbonate concentration was studied with lower amounts of enzyme (2 mg mL^−1^ total protein content in CFEs), with the intention to capture variation in product formation before full conversion can occur. A clear trend was observed in the conversion of 10 mM 1b, with highest product formation obtained with large excess of bicarbonate (55 mM), going in line with observations made with PjCapAB (see ESI, Table S2†).^19^

The ATP concentration was found to correlate well with the amount of product formed, and in general, some uncoupling can be anticipated, as the amount of formed 2b was found inferior to the ATP concentration used in all cases (see Table 4), and a slight excess of ATP was required to reach the highest conversion (*vide infra*).

**Table 4:** Influence of [ATP] on product formation in the PpOplAB-catalyzed hydrolysis of 1b^a^

Entry	[ATP] (mM)	[2b] (mM)	Relative activity (%)
1	Not provided	n.d.	n.a.
2	5	2.7 ± 0.2	36
3	10	5.3 ± 1.4	70
4	12.5	7.6 ± 0.0	100

a Reaction conditions: 4 mg mL^−1^ PpOplAB CFE (total protein content), 10 mM 1b, ammonium bicarbonate buffer (38.8–42.5 mM, pH 8.5), 7.8–8.5 mM MgCl_2_, 30 °C, 900 rpm, 20 h. ATP was added from a stock (100 mM in H_2_O) to final concentration of 5, 10 and 12.5 mM. Mean of triplicates with standard deviation.

### ATP regeneration

**Fig. 4:**
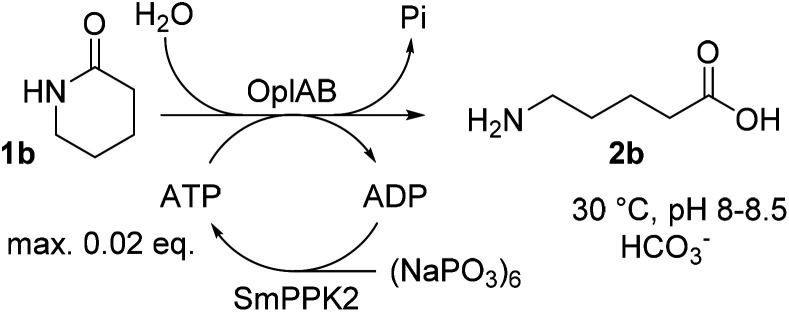
Implementation of an ATP regeneration system based on a polyphosphate kinase 2 (SmPPK2) and Graham's salt (inorganic metaphosphate) in the conversion of 1b by PpOplAB.

ATP is required in the reaction of OplAB on 5-oxo-l-proline^20^ and of CapAB on ε-caprolactam.^18^ An ATP binding site was detected in PjCapA and relevant amino acids involved in binding are conserved in PpOplA.^19^ ATP was proposed to activate the substrate by phosphorylation through the action of subunit A, and is released as ADP, while subunit B hydrolyzes the phosphorylated species in a subsequent step.^19,20^ This explains the requirement for at least stoichiometric amounts of ATP, which is practically not compatible with atom-economic and cost-effective biocatalytic applications. The use of catalytic quantities of ATP for the biocatalytic hydrolysis of lactams was therefore investigated by implementing an enzymatic regeneration system based on polyphosphate kinase 2 from class I (PPK2-I), which catalyzes the monophosphorylation of ADP from cheap inorganic polyphosphate (Fig. 4).^27^ The PPK2-I SMc02148 from *Sinorhizobium meliloti*^28^ was selected because this enzyme was shown compatible with other ATP-dependent enzymatic systems.^29^ The enzyme was over-expressed in *E. coli* (see ESI, Fig. S6†) and added as CFE to the standard reaction mixture supplemented with cheap Graham's salt (*i.e.*, sodium metaphosphate). Full conversion of 10 mM 1b could be observed in the presence of 0.5 mM ATP, 5 mM Graham's salt and 25 mM ammonium bicarbonate (see Table 5). Without the kinase, the conversion was strictly limited to the catalytic amount of ATP present (<5% conversion). Higher amount of polyphosphate lowered the conversion. Under the tested reaction conditions, a clear advantage of using the ATP recycling system could be seen, compared to the addition of excess of ATP, which led to only 31% of the activity obtained with the recycling system (see Table 5, entry 4). Further tests were performed, indicating that 0.1 mM ATP was sufficient to reach full consumption of 10 mM 1b (see Table 6). On 25 mM of 1b, increasing the ATP concentration from 0.1 mM (0.004 eq.) to 0.5 mM (0.02 eq.) had a strong positive impact on conversion and up to 13.9 mM of 2b was formed in the presence of 10 mM Graham's salt (Table 6). A time study indicated that the reaction was particularly rapid during the first 90 min (7.9 mM of 2b formed) and then began to slow down, with maximum conversion reached after about 10 h (see ESI, Fig. S7†). Increasing the substrate concentration to 50 mM barely improved the product formation (14.7 mM of 2b), even in the presence of higher bicarbonate concentration. Collectively, the data indicate that the PpOplAB/SmPPK2 platform can generally sustain high substrate concentrations. Based on densitometric analysis of SDS-PAGE, the amounts of enzyme in the crude lysate were estimated for PpOplAB (3.4 μM) and SmPPK2 (18.1 μM). In the case of the biotransformation of 50 mM 1b that led to the formation of 14.7 mM 2b, PpOplAB achieved a turnover number (TON) of 4360 and SmPPK2, a TON of 785 (see ESI, Table S1†). Both values indicate a good to excellent catalytic efficiency in the cleavage of δ-valerolactam under ATP-regeneration conditions.

**Table 5:** Influence of the reactions conditions in the ATP regeneration system on the conversion of 1b by PpOplAB

Entry	Varying conditions	Relative activity (%)
1	Standard^a^	100^b^
2	No PPK2-I^c^	3
3	20 mM Graham's salt^c^	86
4	No PPK2-I^c^, 12.5 mM ATP (1.25 eq.)	31

a Standard reaction conditions: 4 mg mL^−1^ PpOplAB CFE (total protein content), 10 mg mL^−1^ SmPPK2 CFE (total protein content), 10 mM 1b, 0.5 mM ATP, 5 mM Graham's salt (corresponding to 30 mM monophosphate), 25 mM ammonium bicarbonate, 20 mM NaCl and 2 vol% glycerol (both originating from SmPPK2 lysis buffer), HEPES buffer (50 mM, pH 8.0, ∼8 mM MgCl_2_), 900 rpm, 30 °C, 22 h.

b Full conversion (see ESI, Fig. S36†).

c 10 mM ammonium bicarbonate.

**Table 6:** Conversion of 1b to 2b by PpOplAB with ATP regeneration^a^

Entry	[1b] (mM)	[ATP] (mM) (eq.)	[2b] formed (mM)
1	10	0.1 (0.01)	7.8 ± 0.4^b^
2	10	0.5 (0.05)	7.4 ± 0.1^b^
3	25	0.1 (0.004)	7.9 ± 1.7
4	25	0.5 (0.02)	13.9 ± 3.0
5	50	0.1 (0.002)	11.4 ± 1.0
6	50^c^	0.5 (0.01)	14.7 ± 1.1

a Reaction conditions: 4 mg mL^−1^ PpOplAB CFE (total protein content), 5 mg mL^−1^ SmPPK2 CFE (total protein content), [1b] as indicated, [ATP] as indicated, 10 mM Graham's salt (equivalent to 60 mM monophosphate), 1 vol% glycerol, 10 mM NaCl in ammonium bicarbonate buffer (pH 8.5). Final concentration MgCl_2_: 7.3–9.2 mM and ammonium bicarbonate: 27–37 mM (variations due to different levels of dilution after addition of stock solutions of 1b and ATP in water), 900 rpm, 30 °C, 24 h. Mean of triplicates with standard deviation.

b Full consumption.

c Increasing [NH_4_HCO_3_] to 25 mM did not improve the product formation (data not shown).

**Fig. 5:**
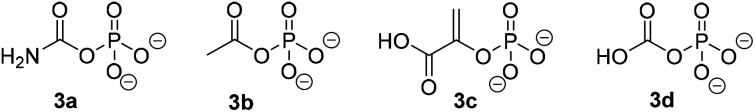
Phosphodonors (3a–3c) tested in place of ATP with PpOplAB and structure of carboxyphosphate 3d.

Similarly to PjCapAB, PpOplAB requires bicarbonate for activity. A direct correlation was seen between the concentration of bicarbonate and the amount of product formed, with highest product concentration obtained in the presence of excess of bicarbonate (*vide supra*, and see ESI, Table S2†). With PjCapAB, a carboxyphosphate formed from bicarbonate and ATP was postulated as a possible intermediate able to phosphorylate the substrate, among various mechanistic proposals.^19^ We therefore hypothesized that other phosphodonors similar to carboxyphosphate could bypass the strict requirement for ATP. A range of alternative phosphodonors (Fig. 5) was explored and added in place of excess of ATP (1.25 eq.). With 2-phosphoenolpyruvate (3c), no product formation could be detected. In contrast, the use of both carbamoyl phosphate (3a) and acetyl phosphate (3b), structurally related to carboxyphosphate 3d, resulted in the formation of 0.3 mM 2b, corresponding to 10% of the activity obtained with the same amounts of ATP (Table 7). PpOplAB is thus catalytically active in the hydrolysis of 1b in absence of ATP and achieved a TON of 176 with the alternative phosphodonors 3a and 3b. These data provide some clues that the structurally related carboxyphosphate may be involved in the first step of the reaction and that under standard conditions, ATP may not be directly phosphorylating the substrate, contrarily to what was postulated for one bacterial 5-oxoprolinase, for which no bicarbonate dependency was reported.^20,25^ This would suggest a role of ATP with PpOplAB similar to the cases of acetone and acetophenone carboxylases, which generate carboxyphosphate as a first intermediate in the catalytic cycle. These enzymes however transfer the carboxy moiety, and not the phosphate, to their respective substrate. The phosphotransfer to the substrate by PpOplAB may not be enzymatic and cases of spontaneous phosphorylation of a range of acceptors with acetyl phosphate have been reported.^30^ Unambiguous is that PpOplA and other homologous subunits are necessary to allow the utilization of ATP as primary source of phosphate *en route* to substrate activation, which exact mechanism remains to be elucidated.

**Table 7:** Influence of the type of phosphodonor in the PpOplAB-catalyzed hydrolysis of 1b^a^

Entry	Phosphodonor	[2b] formed (mM)	Relative activity (%)
1	3a	0.3 ± 0.00	10
2	3b	0.3 ± 0.01	10
3	3c	n.d.	n.a.
4	ATP	2.9 ± 0.2	100

a Reaction conditions: 2 mg mL^−1^ PpOplAB CFE (total protein content), 10 mM 1b, 12.5 mM phosphate donor, 5 mM ammonium bicarbonate in HEPES buffer (50 mM, pH 8.0, 7.8 mM MgCl_2_), 30 °C, 900 rpm, 20 h. Mean of triplicates with standard deviation. See ESI, Fig. S35.†

### ^31^P-NMR

Given that structures similar to carboxyphosphate can trigger hydrolysis of the lactam by PpOplAB in the absence of ATP, it appears likely that the role of ATP is to generate a phosphorylated intermediate species for activation of the substrate. While phosphotransfer from carboxyphosphate to the substrate has been proposed to happen, a carboxylation of the oxygen of the tautomeric form of the lactam, the lactim, to a carbonate cannot be ruled out. This would go in line with the carboxylating role of carboxyphosphate in a number of enzymatic reactions.^21–23^ The resulting carbonate monoester would then be hydrolyzed by PpOplB. Further investigations of the mechanism were attempted by ^31^P-NMR, with the aim to reveal the formation of phosphorylated intermediate species. The reaction mixture of the biotransformation of 1b in the presence of excess of ATP (12.5 mM) under conditions similar to those reported in Table 3 was analyzed in parallel by LC-MS. ^31^P-NMR allows to discriminate between most phosphorylated species, especially when different numbers of phosphate units are involved, and can be used to easily monitor the conversion of ATP to ADP. After 1 h reaction catalyzed by PpOplAB, 2 mM of 2b was formed and a ratio of ATP/ADP of 1 : 2.28 was measured (see Table 8 and ESI, Fig. S40 and S41†), indicating 70% hydrolysis of ATP, which corresponds to 8.7 mM of ADP formed. Taking into consideration the formation of 2.0 mM of product, the uncoupling of ATP therefore reaches 77%. This highlights the importance of excess of ATP to achieve high conversion levels. Noteworthy is that ATP could be hydrolyzed to ADP in absence of lactam (see Table 8, entry 2). This goes in line with previous observations with PjCapAB, which catalyzes futile hydrolysis of ATP.^19^ A higher ATP hydrolysis compared to the amount of glutamate formed was also observed in early studies on bacterial 5-oxo-l-prolinase.^20^ In the case of the *N*-methylhydantoinase system from *Pseudomonas putida* 77,^31^ γ-butyrolactam and δ-valerolactam could trigger the consumption of ATP, however hydrolysis to the amino acid did not take place, indicating that substrate selectivity is very much subunit and enzyme dependent.

**Table 8:** Correlation between hydrolysis of ATP to ADP and hydrolysis of 1b to 2b^a^

Entry	Conditions	[2b]^b^ (mM)	ATP/ADP^c^	[ADP]^d^ (mM)	Uncoupling^e^ (%)
1	10 mM 1b	2.0	1 : 2.28	8.7	77
2	No substrate	n.a.	1 : 0.60	4.7	37

a Reaction conditions: 1 mg mL^−1^ PpOplA/B CFE (total protein content), 12.5 mM ATP, ammonium bicarbonate buffer (39 mM, pH 8.5), 8 mM MgCl_2_, 30 °C, 900 rpm, 1 h.

b Amount of formed 2b quantified by HPLC-MS.

c Ratio obtained by ^31^P-NMR analysis.

d Obtained based on the measured ratio of ATP/ADP.

e Corresponds to the percentage of ADP formed by ATP hydrolysis and not by phosphorylation of 1b, value obtained by ([ADP] − [2b])/[ADP] × 100.

Finally, during our studies, ^31^P-NMR analysis did not allow to discriminate between potential further phosphorylated intermediates during the reaction.

## Experimental

### Procedures for biotransformations

#### Representative biotransformations using CFEs of PpOplA and PpOplB expressed individually

The cell pellets of PpOplA and PpOplB obtained by individual overexpression were resuspended separately in lysis buffer (ammonium bicarbonate, 50 mM, pH 8.5 containing 4 mM MgCl_2_) and disrupted by sonication. The cell debris were removed by centrifugation (17 000 rpm, 30 min, 4 °C) and the protein concentration of PpOplA and PpOplB was measured in the CFEs by the Bradford assay. Reactions were performed in 2 mL micro reaction tubes in ammonium bicarbonate buffer (50 mM, pH 8.5) containing MgCl_2_ (4 mM), CFEs of PpOplA and PpOplB (11.4 mg mL^−1^ each, total protein content), ATP and lactam in concentrations as indicated, in a final volume of 1 mL. The samples were incubated with horizontal shaking for 16 h at 30 °C and 140 rpm. Two control reactions were run in parallel in the absence of (i) enzyme and (ii) substrate. After this time, the mixtures were filtered by centrifugation and the supernatant was split into two 2 mL micro reaction tubes (each 450 μL). One sample was lyophilized to be used for amino acid derivatization and further measurements, and the other tube was used for lactam extraction, as reported in the ESI.†

#### Representative biotransformations using CFEs of co-expressed PpOplAB and ATP-regeneration system with a kinase

The cell pellets containing PpOplAB were resuspended in 6 mL ammonium bicarbonate buffer (50 mM, pH 8.5, 10 mM MgCl_2_) per g cell mass, the cells were disrupted by sonication and cell debris were removed by centrifugation (18 000 rpm, 30 min, 4 °C) to deliver CFEs. Biotransformations were performed on an analytical scale (500 μL if not stated otherwise) in 1.5 mL micro reaction tubes. The reactions were run in ammonium bicarbonate buffer (pH 8.5, 27–37 mM) containing 10 mM NaCl and MgCl_2_ (final concentration 7.3–9.2 mM), 4 mg mL^−1^ PpOplAB CFE (total protein content), 5 mg mL^−1^ SmPPK2 CFE (total protein content), lactam and ATP in concentrations as indicated, 10 mM Graham's salt (equivalent to 60 mM monophosphate), 1 vol% glycerol. The samples were incubated in an Eppendorf Thermomixer at 900 rpm, 30 °C, 24 h. 25 μL reaction volume were used for analysis by LC-MS, as detailed in the ESI.†

## Conclusions

The chemical stability of δ-valerolactams could be efficiently circumvented and enzymatic hydrolysis to the ω-amino acids proceeded under mild reaction conditions in aqueous medium at the expense of ATP in presence of bicarbonate. The bacterial system PpOplAB likely utilizes the lactim tautomer of the lactam as reactive species, taking advantage of the significant partial C–N double bond character of the amide bond responsible for the resonance stabilization of the δ-lactams. A kinase-based ATP regeneration with inexpensive Graham's salt was successfully implemented, and is especially advantageous given the substantial ATP uncoupling observed during lactam hydrolysis, which leads to futile hydrolysis of ATP. Although the intermediate species involved in the reaction were not identified, the fact that ATP could be replaced by the alternative phosphodonors carbamoyl phosphate and acetyl phosphate provides hints that the role of bicarbonate in the reaction is to generate a carboxyphosphate intermediate for substrate activation. PpOplAB is not effective in the hydrolysis of γ-butyrolactam. However, the trace activity observed is promising and suggests that the chemical stability of this molecule may be tackled in the future using biological tools.

## Conflicts of interest

The authors declare that there are no conflicts of interest.

## Supplementary Material

GC-026-D3GC04434C-s001
