# Supplementary material for: Mild hydrolysis of chemically stable valerolactams by a biocatalytic ATP-dependent system fueled by metaphosphate
Source: Green Chem. 2023 Dec 7;26(8):4498–505. doi: 10.1039/d3gc04434c (PMC11033972; doi:10.1039/d3gc04434c)
Supplement: GC-026-D3GC04434C-s001 [file GC-026-D3GC04434C-s001.pdf]

Electronic Supplementary Material (ESI) for Green Chemistry.
This journal is © The Royal Society of Chemistry 2023

Mild Hydrolysis of Chemically Stable Valerolactams by a Biocatalytic ATP-dependent System Fueled by Metaphosphate

Sebastian Roth,†[a] Somayyeh Gandomkar,†[a] Federico Rossi[a] and Mélanie Hall*[a,b]

[a] Institute of Chemistry, University of Graz, Heinrichstrasse 28, 8010 Graz, Austria
[b] BioHealth, University of Graz
*Email: melanie.hall@uni-graz.at
† equal contributions

Table of content

1. Individual cloning of OplA and OplB p.2
1.1. Expression at 30 °C after induction with IPTG p.2
1.2. Expression by autoinduction p.3
1.3. Purification of individually expressed PpOplA and PpOplB p.4
2. Co-expression of PpOplA and PpOplB p.5
2.1. Cloning p.5
2.2. Overexpression and preparation of cell-free extracts in ammonium bicarbonate p.7
2.3. Purification of PpOplAB p.7
2.4. Preparation of cell-free extracts of PpOplAB without ammonium bicarbonate p.7
3. Access to polyphosphate kinase SmPPK2 (Uniprot Q92SA6) p.8
4. Densitometric analysis for protein content in CFE p.8
5. Procedures for biotransformations p.9
5.1. Biocatalytic reactions using CFEs of PpOplA and PpOplB expressed individually p.9
5.2. Biocatalytic reactions using lyophilized cells of PpOplA & PpOplB expressed individually p.9
5.3. Biotransformations with CFEs of co-expressed PpOplAB p.9
6. Influence of [NH_4HCO_3] on product formation in the conversion of 1b by CFE of PpOplAB p.10
7. Time study of the conversion of 1b with CFE of PpOplAB p.10
8. Synthesis of reference amino acids p.10
9. Analytical methods p.11
9.1. Gas chromatography & mass spectrometry p.11
9.1.1. Derivatization p.11
9.1.2. Calibration curves for GC-FID p.11
9.1.3. Representative GC-FID chromatograms p.15
9.2. HPLC-MS p.16
9.2.1. Conditions p.16
9.2.2. Calibration curves for HPLC-MS p.17
9.2.3. Representative chromatograms p.19
10. Analysis of enantioselectivity p.28
11. NMR p.31
11.1. ^{31}P-NMR p.31
11.2. ^{1}H-NMR p.32
12. Safety concerns p.34
References p.34

1. Individual cloning of OplA and OplB

The synthetic gene coding for 5-oxoprolinase A (OplA) from *Pseudomonas putida KT2440* with Uniprot accession number Q88H50 flanked with *Nde*I at *N*-terminus and *Hind*III at *C*-terminus restriction sites and codon optimized for expression in *E. coli* and the gene coding for 5-oxoprolinase B (OplB) from *Pseudomonas putida KT2440* with Uniprot accession number Q88H51 flanked with *Nde*I at *N*-terminus and *Hind*III at *C*-terminus restriction sites and codon optimized for expression in *E. coli* were ordered and cloned in pET28a(+) using standard molecular biology protocols. Transformation in competent cells E. coli NEB5α was followed by transformation in *E. coli* BL21(DE3).

1.1. Expression at 30 °C after induction with IPTG

Overnight cultures were prepared form the glycerol stocks in 250 mL baffled autoclaved shaking flasks. 100 mL LB medium were mixed with 100 µL of 50 mg/mL kanamycin stock solution (50 µg/mL final concentration), and 300 µL of glycerol stock of PpOplA or PpOplB. The flasks were incubated at 37 °C and 120 rpm overnight. For growing the cells, 2 L baffled shaking flasks were used. Each flask was filled with 500 mL of autoclaved LB medium, 500 µL of 50 mg/mL kanamycin stock solution (50 µg/mL final concentration) and 5 mL of overnight culture. Incubation was done at 37 °C and 120 rpm. At an OD_{600} of 0.6, 500 µL of 1 M IPTG stock (1 mM final concentration) was added to each flask and incubation was done at 30 °C and 120 rpm overnight. The next day, the cells were harvested by centrifugation (8000 rpm, 20 min, 4 °C) and washed two times with dH_2O.
For determination of over-expression level, the pellets were resuspended in lysis buffer (50 mM sodium phosphate buffer containing 300 mM NaCl, 8% glycerol, 10 mM imidazole, pH 7.5, 10 mL buffer per g of pellet). Then the cells were disrupted by ultrasonicating the suspension on ice [(30% amplitude, 2 sec pulse on, 4 sec pulse off for 5:30 min) x 2]. Afterwards the centrifugation was done (18000 rpm, 20 min, 4 °C) to remove cell debris. The Bradford assay was used to measure the protein concentration of each sample. The expression level of PpOplA and PpOplB was analyzed from the supernatant and the pellet samples by SDS-PAGE (10% SDS-gels, using MOPS as running buffer, 15 µg protein loading on each cavity, Figure S1). The molecular weight of the PpOplA is about 75 kDa and PpOplB is about 63 kDa.

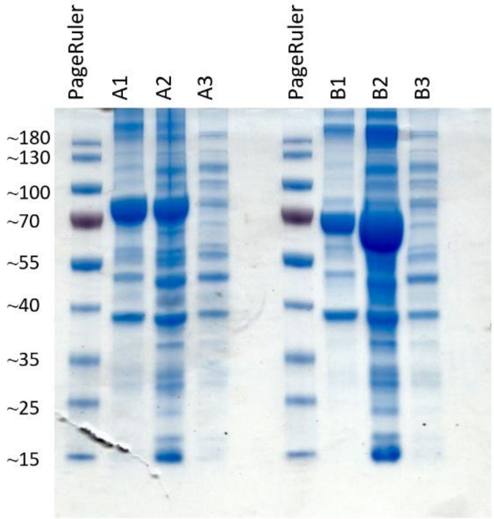

Figure S1. SDS-PAGE from expression of PpOplA and PpOplB at 30°C. Lane A1: Pellets from PpOplA after ultrasonication, Lane A2: Pellets from PpOplA before ultrasonication, Lane A3: CFE from PpOplA, Lane B1: Pellets from PpOplB after ultrasonication, Lane B2: Pellets from PpOplB before ultrasonication, Lane B3: CFE from PpOplB.

1.2. Expression by autoinduction

PpOplA and PpOplB were expressed using autoinduction medium. The following solutions were prepared: 20x NPS containing 900 mL dd H_2O, 66 g $(NH_4)_2SO_4$, 136 g KH_2PO_4 and 142 g Na_2HPO_4 and 50x 5052 containing 250 g glycerol, 700 mL dd H_2O, 25 g glucose and 100 g α-lactose monohydrate. Mixing was done in the given sequence and after stirring and heating for dissolving the components, the volume was set to 1 L. Afterwards, 20x NPS solution was autoclaved and 50x 5052 solution was filter sterilized using 0.22 µm syringe filters under laminar flow. Solutions of 40% glucose, 1 M $MgSO_4$ and 1000x trace metal mixture were prepared and filter sterilized using 0.22 µm syringe filters under laminar flow. The 1000x trace metal mixture was containing 50 mM $FeCl_3$, 20 mM $CaCl_2$, 10 mM $MnCl_2$ and 10 mM $ZnSO_4$, and 2 mM $CoCl_2$, 2 mM $CuCl_2$, 2 mM $NiCl_2$, 2 mM Na_2MoO_4, 2 mM Na_2SeO_3, and 2 mM H_3BO_3. The trace metal mix was assembled from autoclaved stock solutions of the individual components except for $FeCl_3$, which was added from the 0.1 M solution in ~0.12 M HCl.

For preparation of pre-culture, 4.635 mL of LB medium was mixed with 5 µL of 1 M $MgSO_4$, 100 µL of 40% glucose, 250 µL of 20x NPS and 10 µL of 50 mg/mL kanamycin stock solution, then 2 mL of this mixture was transferred into a 15 mL falcon Sarstedt tube and 5 µL of glycerol stocks from PpOplA or PpOplB was added to this mixture then incubation was done at 37 °C and 120 rpm overnight.

For the main culture, 926 mL of LB medium was mixed with 2 mL of 1 M $MgSO_4$, 20 mL of 50x 5052, 50 mL of 20x NPS, 2 mL of 50 mg/mL kanamycin stock solution and 200 µL of trace metal solution. Then 450 mL of this mixture was transferred into a 2 L baffled flask and 900 µL from pre-culture of PpOplA or PpOplB was added. Incubation was done for 48 h, 17 °C and 120 rpm. After 48 h, the cells were harvested by centrifugation (8000 rpm, 20 min, 4 °C) and washed with Tris-HCl (100 mM, pH 7.5 containing 4 mM $MgCl_2$). 3.5 g of the fresh pellets were resuspended in ammonium bicarbonate buffer (50 mM, pH 8.5, containing 4 mM $MgCl_2$, 10 mL buffer per g pellet). Then the cells were disrupted by ultrasonicating the suspension on ice [(30% amplitude, 1 sec pulse on, 4 sec pulse off for 2:30 min) x 2]. After centrifugation (18000 rpm, 20 min, 4 °C), the cell free extracts (CFE) were obtained. The expression level of PpOplA and PpOplB was analyzed by SDS-PAGE (10% SDS-gels, using MOPS as running buffer, 15 µg protein loading on each cavity, Figure S2).

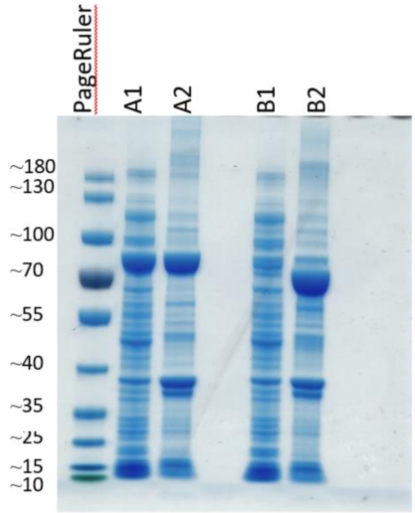

Figure S2. SDS-PAGE from autoinduction expression of PpOplA and PpOplB. Lane A1: CFE of PpOplA after ultrasonication in ammonium bicarbonate buffer, Lane A2: Pellets from PpOplA after ultrasonication in ammonium bicarbonate buffer, Lane B1: CFE of PpOplB after ultrasonication in ammonium bicarbonate buffer, Lane B2: Pellets from PpOplB after ultrasonication in ammonium bicarbonate buffer.

In case of resuspension of the cell pellet in Tris-HCl (100 mM, pH 7.5 containing 4 mM $MgCl_2$), low recovery of soluble OplA was observed:

After 48 h, the cells were harvested by centrifugation (8000 rpm, 20 min, 4 °C) and washed with Tris-HCl (100 mM, pH 7.5 containing 4 mM $MgCl_2$). 200 mg of the pellets were taken up into 1.5 mL micro reaction tubes and resuspended in 800 µL Tris-HCl (100 mM, pH 7.5 containing 4 mM $MgCl_2$). Then the cells were disrupted by ultrasonicating the suspension on ice (30% amplitude, 2 sec pulse on, 4 sec pulse off for 5:30 min). Afterwards the centrifugation was done with bench top centrifuge at room temperature (1460 rpm, 2 x 5 min) to remove the cell debris. Then SDS-PAGE from the supernatant and the pellet samples of PpOplA and PpOplB was done (Figure S3).

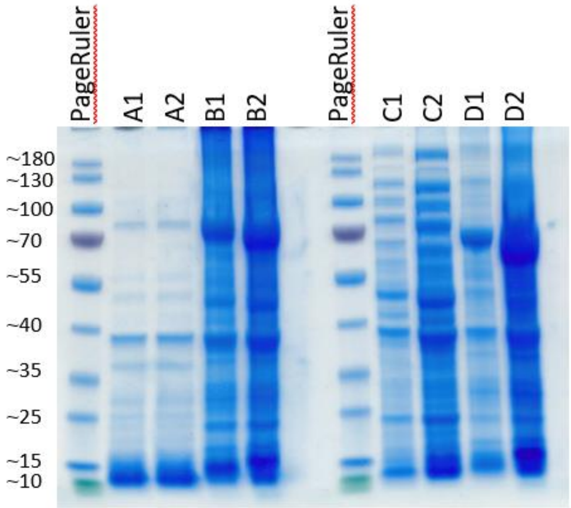

Figure S3. SDS-PAGE from autoinduction expression of PpOplA and PpOplB. Lane A1: CFE of PpOplA after ultrasonication (15 µg protein loading), Lane A2: CFE of PpOplA after ultrasonication (>15 µg protein loading), Lane B1: Pellets from PpOplA after ultrasonication (15 µg protein loading), Lane B2: Pellets from PpOplA after ultrasonication (>15 µg protein loading), Lane C1: CFE of PpOplB after ultrasonication (15 µg protein loading), Lane C2: CFE of PpOplB after ultrasonication (>15 µg protein loading), Lane D1: Pellets from PpOplB after ultrasonication (15 µg protein loading), Lane D2: Pellets from PpOplB after ultrasonication (>15 µg protein loading).

1.3. Purification of individually expressed PpOplA and PpOplB

For the purification, all the buffers were filtered using 0.2 µm filter papers and degassed before using. To purify the *Pp*OplA and *Pp*OplB, the pellets obtained from autoinduction expression were resuspended in the lysis buffer (ammonium bicarbonate, 50 mM, pH 8.5, containing 300 mM NaCl, 4 mM $MgCl_2$, 10 mM imidazole and 8% glycerol, 10 mL buffer per g of pellet). Then the pellets were disrupted by ultrasonication [(30% amplitude, 1 sec pulse on, 4 sec pulse off for 2:30 min) x 2]. Afterwards the cell debris was removed with centrifugation (17000 rpm, 30 min, 4 °C). The supernatants from *Pp*OplA and *Pp*OplB were separately filter sterilized using a 0.45 µm syringe filter. The enzymes were purified separately using 5 mL His-Trap™ FF columns. The column was washed first with 50 mL ddH_2O then with 50 mL binding buffer (ammonium bicarbonate, 50 mM, pH 8.5, containing 300 mM NaCl, 50 mM imidazole and 8% glycerol). Afterwards the sample was loaded onto the column and washed again with 50 mL binding buffer to remove protein impurities. The elution of the enzyme was done using 10 to 15 mL of elution buffer (ammonium bicarbonate, 50 mM, pH 8.5, containing 300 mM NaCl, 250 mM imidazole and 8% glycerol). The Bradford assay was done from all the collected

fractions from *Pp*OplA and *Pp*OplB purification and SDS-PAGE was run from these samples (Figure S4). The eluted fractions were concentrated using VIVASPIN tubes (MWCO 10 kDa, 4,688 x g at 4 °C). After concentration, the samples were desalted by using PD-10 desalting columns and eluted with ammonium bicarbonate buffer (50 mM, pH 8.5, containing 4 mM $MgCl_2$). The purified enzymes were directly used in the biotransformation reactions and the leftovers were stored at 4 °C.

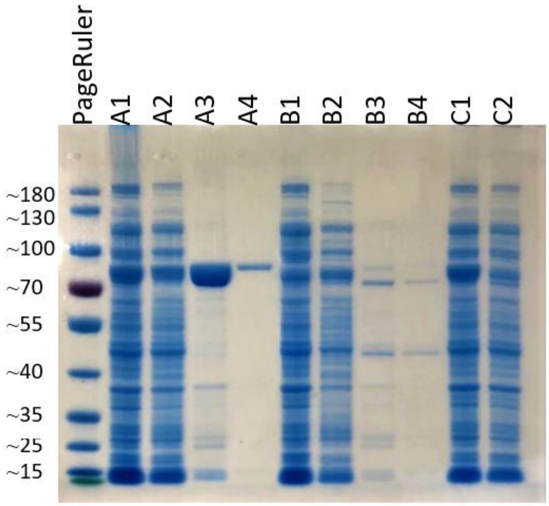

Figure S4. SDS-PAGE from purification of PpOplA and PpOplB. Lane A1: Flow-through from PpOplA, Lane A2: Washing fraction from PpOplA, Lane A3: Elution fraction 1 from PpOplA, Lane A4: Elution fraction 2 from PpOplA, Lane B1: Flow-through from PpOplB, Lane B2: Washing fraction from PpOplB, Lane B3: Elution fraction 1 from PpOplB, Lane B4: Elution fraction 2 from PpOplB, Lane C1: CFE from PpOplA, Lane C3: CFE from PpOplB.

2. Co-expression of PpOplA and PpOplB

2.1. Cloning

For co-expression of PpOplA and PpOplB, the pETDuet-1 vector was used. Both genes were codon-optimized for expression in *E. coli*. The plasmid was obtained from Biocat (Heidelberg, Germany) with PpOplA cloned into multiple cloning site I (BamHI and *Hind*III) providing the N-terminally His-tagged enzyme. PpOplB was cloned into multiple cloning site II (*Nde*I and *Xho*I).

PpOplA DNA sequence (restriction site underlined)

acagccaggatccG**ATG**CGCAATCAGTATCGCCTGGGTGTTGATGCAGGTGGTACCTTCACCGACTTCATTCTGGC
CGATCGCGATGGCGGCGTTCAGCTGTTCAAAGCCCCGAGTACCCCGCAGGATGGCACCCTGGCAATTCGCGCC
GGTCTGGCACAGATTGCAGATGCCACCGGTCGCAGCCCGGCCGAACTGATTGCAAATTGTGATCTGTGTATTA
ATGGCACCACCGTTGCACTGAATGCACTGATTGAACGCACCGGTGTGAAAGTTGGTCTGCTGTGTACCGAAGG
CCATGAAGATAGTCTGGAAATTCGCCTGGGTCATAAAGAAGAAGGCCATCGTTATGATGCCCATTATCCGCCG
GCACACATGCTGGCCCCGCGCCACTTACGCCGCCCTATTGGTGGTCGTATTCTGGGCGATGGCCGTGAACATA
GCCCGCTGGATGAAGCCGCCATTCATGCAGCAATTGATTACTTCCGCGCAGAAGGCGTTCAGGCAGTGGCCAT
TAGCTTCCTGTGGAGTGTTCGTAATCCGAGCCATGAACAGCGTGCAGCAGAACTGGTTCGCGCCGCACTGCCG
GAAGTGTTCGTGTGTACCGGTTGCGAAGTGTTCCCGCAGATTCGCGAATATACCCGCACCAGCACCACCGTTG
TGAATGCCTATCTGAGTCCGGTGATGGCACGCTATGTTGCCCGTATTGATAATCTGTTCCAGGAACTGGGTGCA
CAGCAGCCGGTGCGTTACTTCCAGAGTAATGGTGGCCTGGCACCGGGTCATCTGATGCGTGAACGCGCAGTTA

ATGCAATTAATAGTGGTCCGGCAAGCGCCCCGCAGGCAGGTCTTGCAGTTGCCCGCCCGTTCGGCATTGATAA
TGTGATTACCGTTGATATGGGCGGCACCAGCTTCGATATTACCCTGACCAATGCAGGCCGCACCAACTTCAGCA
AAGATGTTGACTTCCTGCGCCAGCGCATTGGTGTGCCGATGATTCAGGTTGAAACCTTAGGCGCAGGCGGCGG
TAGTATTGCACATCTGGATGAATTCGGCATGCTGCAGGTGGGCCCGCGTAGCGCAGGTGCAATGCCGGGTCC
GGTGTGTTATGGTAAAGGCGGTCGTGAACCGACCGTGACCGATGCCAATCTGGCACTGGGCTATCTGCCGGAT
GGCGCACTGCTGGGTGGTAGCATTCGTCTGAATCGTCAGGCAGCACTGGATGCAATTCGCAGTAAAATTGCCG
AACCGCTGGGCATTAGTGTGGAACGTGCCGCCTTCGGCATTACCACCCTGGTGAATCTGAATATGGTGAATGG
TATTCGTCGTGTGAGCATTGAACGCGGTCATGATCCGCGTGACTTCGCACTGATTGGTGCCGGTGGCGCCGCC
GGTATGCATGTTGTTCGTCTGGCAGAAGAAATTGGCATGCATACCGTGCTGATTCCGAAAGTTGCAAGTGGTC
TGTGTGCCTTCGGCCAGATTCTGAGTGATGTTCGCTATGATCAGCTGACCAGCCTGAGCATGCGTCTGGATGCA
GGCCATGTTGATCTGGCCCAGCTGAATCAGGCCCTGGCAGAACTGCGTCAGCAGGGTCTGGCCAATCTGCGCG
AAGATGGCTTCGGCGATCAGGCAAGTAGTTGTCATTATACCCTGGAAATGCGTTATCTGGGTCAGATTCATGA
ATGCAGCGTTGATCTGCAGCAGCATAGCCTGGATGAAGCAGGCCTGGCCGCCCTGATTAGCCGCTTCCATAGT
CGTCATCAGACCCTGTATAGTTATAGTGAACCGGCCAGCCCGGTTGAACTGGTTAATCTGGAATGCAGTGTTAT
TGGCCATCTGCCGCGTCCGCCGCAGCCGGAGTTACAGGGTCCGGCTGAACCGCCGGCCCCGACCGCACAGAG
TGTGAGACCGATGCTGTTCAGTGCCGATGGCGAATGGCAGCCGACCCCGGTGTTCAATGGCAATCATCTGCTG
CCGGGCCAGACCGTGCATGGTCCGTGCGTTATTGAAGAAGATACCACCAATATTGTGCTGCCGCCGGGTTGGC
AGGCCCGCTTAGAACCGAGCGCCACCTATCGTGTGACCCGTGGTGGT**TAA**aagcttgcgg

PpOplB DNA sequence (restriction site underlined)

gatatacat**ATG**CAGACCGTTGATCCGATTACACTGGCAGTTGTTCGTGGTGCACTGGAAACCGCACAGCGTGAA
ATGACCCTGACACTGGAAAAAACCGGTCGTAGCAGCGTTTTTAATCTGGCACATGATTATAGCAACAGCCTGTT
TGATCATCTGCCGGAAATGATTCTGCAGGGTCAAGATATTCCGATTCATCTGGGTAGCCTGATTCCGGCAATGA
AATGTGTTGCAGAATTTTTTGGTGATGATATTGCCGAAGGCGACGTGATTTATCATAATGATCCGGCATATAAA
GGCAGCCATATTCTGGATTGTTGCATGTATAAACCGGTGTATTATCAGGATGAACTGGTTTTTTGGACCGTGTG
TAAAGGTCATCTGACCGATATTGGTGGTCCGGTTCCGGCAGGTTATAATCCGGATGCAAAAGAAATTTATGCA
GAAGGTCTGCGTATTCCGCCTGTTAAACTGTGGGAAAAAGGTAAACGTCGTGAAGATGTGATCAACTTTATGC
TGACCAATATGCGTGCACGTCCGTATCAAGAAGGTGATCTGAACGCACAGTATGGTGCATGTAAAGTTGGTGA
ACGTCATCTGCTGGAACTGCTGGATAAATATGGTGTTGCACAGGTTCGTGCATGTATTGCAGAACTGAAAAAT
ATGGCAGATCGTCACATGCGTGCCCTGCTGCGTGATGTTCCGGATGGTGATTATAGTGGCACCGCAGTTCTGG
AAGATGCAGGTCATGGTCTGGGTGAACTGGCAATTACCGCACATGTTCAGATTCGTGGTGATCAGGCCCATGT
TCGTATTGAAAGCCCTCCGCAGGTTCCGTATTTCATTAATAGCTATGAAGGTAATAGCGTGAGCGGTGTTTATC
TGGGTCTGATGATGTTTGCCCAGGTTGCACCGCCTTATAATGAAGGTCTGTATCGTTGTGTTACCGTTGATGTT
GGTCCGCGTGGCACCCTGTGTAATGCCGAAGAACCGGCACCGCATGTTAATTGTACCACCACACCGATGGAAA
CCCTGGCAGATGCAGTTCGTACCGCACTGGAACAGGCAAGTCCGCAGCGTGTTACCGCAAGCTGGGGTCATA
GCAGCGGTATTAACATTGCAGGTCGTGATCCGCGTAATGGCAATAGCGAATATGTTACCATGGTTCTGGCAAG
CATTATTAGCGGTGCGGGTGCAAATAAAGTTATGGATGGTTGGCATGCATGTGGTCCGCTGTGTTGTTTTGGT
GCCCTGATGAGCGGTGATATTGAACTGTTAGAACATGCATATCCGGTGCTGATTCATCGTTATAGCCTGATGGC
AGATAGCGGTGGTGCCGGTGAACTGCGTGGTGGTAGCGGCACCCGTCTGGAAATTGAACCGCTGGATCATGC
AATGACCGTTGTTGGTTTTGGCGAAGGTCGTCAGCTGCCGACAGCCGGTGCAGCGGGTGCACGTAATGCCTTA
CTGGAACCGAAACTGGGTCGTCTGATCCATCGTCATGCAGATGGTCGCGAAGATCATTATACCCAGAATCCGA
TGCTGACACTGCAGCCTGGTGAACGCATTATCAACATTAATCCAGGTGGTGGTGGTTATGGTGATCCGCTGCG
TCGTCGTGTTGCAGCCGTTTTAGAAGATGTTCGTAATGGTCTGGTTAGTCCGCAGGGTGCAGCACTGGAATAT
GGCGTTGTTCTGGATGCCGATGGTCAGCTGAATGAAACCGCAACACGTCTGGCACGTGCACCGCGTTAAAAGC
TT**TAA**ctcgag

2.2. Overexpression and preparation of cell-free extracts in ammonium bicarbonate

E. coli BL21 (DE3) cells were used and the expression plasmid pETDuet-1_PpOplAB (pEG 732) was introduced via heat shock transformation. An overnight culture with 10 mL of LB medium (100 µg/mL ampicillin) was inoculated with a single colony and incubated at 37 °C and 120 rpm. 500 mL TB medium (100 µg/mL ampicillin) in a 2 L baffled flask were inoculated with 5 mL overnight culture and incubated at 24 °C and 120 rpm for 24 h. The autoinducing properties of TB medium resulted in overexpression of PpOplAB. Cultures were harvested by centrifugation (20 min, 12000 x g, 4 °C), resuspended in ammonium bicarbonate buffer (50 mM, pH 8.5, 10 mM $MgCl_2$) and again centrifuged (45 min, 3166 × g, 4 °C). The resulting pellets were resuspended in 6 mL ammonium bicarbonate buffer (50 mM, pH 8.5, 10 mM $MgCl_2$) per g cell mass, the cells were disrupted by sonication (Branson Ultrasonics™ Sonifier™ SFX250, 30% amplitude, 1 sec pulse on, 4 sec pulse off, total on 2:30 min, two times), and cell debris were removed by centrifugation (30 min, 18000 rpm, 4 °C) to deliver CFEs.

2.3. Purification of PpOplAB

Protein purification by Ni-NTA affinity chromatography was performed using an ÄKTA chromatography system and a HisTrap HP 5 mL column with ammonium bicarbonate buffer (50 mM, pH 8.5, 10 mM $MgCl_2$, buffer A) and ammonium bicarbonate buffer (50 mM, pH 8.5, 10 mM $MgCl_2$, 300 mM imidazole, buffer B). The column was equilibrated with 8% buffer B (5 CV) and the lysate was applied. After washing with 8% buffer B (4 CV), bound PpOplAB was eluted with 100% buffer B (4 CV). The enzyme-containing fractions were pooled and concentrated before being desalted on a PD–10 column. PpOplAB was obtained in ammonium bicarbonate buffer (50 mM, pH 8.5, 10 mM $MgCl_2$) and protein concentration was determined by Bradford assay. Cell lysis and purification process were analyzed via SDS–polyacrylamide gel electrophoresis (main text, Figure 1).

2.4. Preparation of cell-free extracts of PpOplAB without ammonium bicarbonate

Tris-HCl buffer (50 mM, pH 8.0, 10 mM $MgCl_2$) and HEPES buffer (50 mM, pH 8.0, 10 mM $MgCl_2$) were tested as lysis buffer (6 mL per g cell pellet). The lysates were analyzed by SDS-PAGE (Figure S5) and compared to a sample of lysate in ammonium bicarbonate buffer (50 mM, pH 8.5, 10 mM $MgCl_2$). PpOplAB was found to a similar extent in the soluble fractions in Tris-HCl buffer (50 mM, pH 8.0, 10 mM $MgCl_2$) and HEPES buffer (50 mM, pH 8.0, 10 mM $MgCl_2$) while more concentrated samples were obtained in ammonium bicarbonate buffer (50 mM, pH 8.5, 10 mM $MgCl_2$).

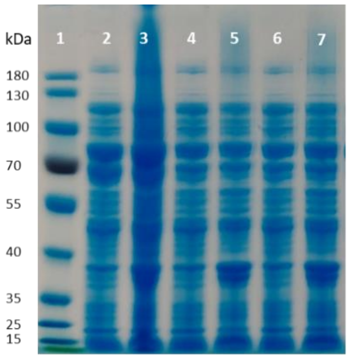

Figure S5. Polyacrylamide gel to analyze PpOplAB lysates (PpOplA 76 kDa, PpOplB 63 kDa) in different buffers. Lane 1: Marker: PageRuler™ Prestained Protein ladder; lanes 2 and 3: lysate and pellet obtained after lysis in ammonium bicarbonate buffer (50 mM, pH 8.5, 10 mM $MgCl_2$); lanes 4 and 5: lysate and pellet obtained after lysis in Tris-HCl buffer (50 mM, pH 8.0, 10 mM $MgCl_2$); lanes 6 and 7:

lysate and pellet obtained after lysis in HEPES buffer (50 mM, pH 8.0, 10 mM $MgCl_2$). Protein loading 35 µg except of lane 3 (unknown concentration).

3. Access to polyphosphate kinase SmPPK2 (Uniprot Q92SA6)

For expression of SmPPK2, *E. coli* BL21 (DE3) cells were used and the plasmid (kind gift from Prof. Yakunin)[1] was introduced via heat shock transformation. An ONC with 10 mL of LB medium (100 µg/mL ampicillin) was inoculated with a single colony and incubated at 37 °C and 120 rpm. 500 mL LB medium (100 µg/mL ampicillin) in a 2 L baffled flask were inoculated with 5 mL overnight culture and incubated at 37 °C and 120 rpm until an OD_{600} of 0.5–0.7. Overexpression was induced using IPTG (0.2 mM) and the culture was incubated for 20–22 h at 20 °C and 120 rpm. Cultures were harvested by centrifugation (20 min, 12000 x g, 4 °C), resuspended in HEPES buffer (50 mM, pH 8.0, 10 mM $MgCl_2$) and again centrifuged (45 min, 3166 x g, 4 °C). The resulting pellets were resuspended in 6 mL HEPES buffer (50 mM, pH 8.0, 10 mM $MgCl_2$, 50 mM NaCl, 5 vol% glycerol) per g cell mass, the cells were disrupted by sonication (Branson Ultrasonics™ Sonifier™ SFX250, 30% amplitude, 1 sec pulse on, 4 sec pulse off, total on 2:30 min, two times), and cell debris were removed by centrifugation (30 min, 18000 rpm, 4 °C). Overexpression was analyzed on SDS-PAGE (Figure S6).

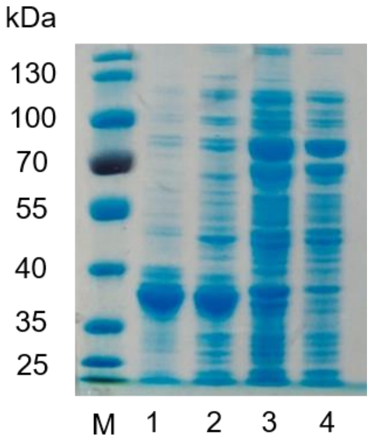

Figure S6. Polyacrylamide gel of SmPPK2 (37 kDa) in HEPES buffer (50 mM, pH 8.0, 10 mM $MgCl_2$, 50 mM NaCl, 5% glycerol) and PpOplAB (76 kDa and 63 kDa) in ammonium bicarbonate buffer (50 mM, pH 8.5, 10 mM $MgCl_2$): M: PageRuler™ Prestained Protein Ladder; lane 1: pellet and lane 2: lysate of SmPPK2; lane 3: pellet and lane 4: lysate of PpOplAB. 15 µg protein loading in lanes 2 and 4, which were used for densitometry, see below.

4. Densitometric analysis for protein content in CFE

The software ImageJ was used to compare the pixel density of the total protein lane (15 µg applied on SDS gel) with the spots of the target enzymes. A photograph of the SDS gel was opened with ImageJ and the image was first changed to black and white (Image→Type→32-bit) and then inverted (Edit→Invert Image), which reduces the picture to bright and dark elements. The whole lanes were selected and their integrated density values were measured (Table S1). The process was repeated for the bands corresponding to SmPPK2, PpOplA and PpOplB.

Table S1. Estimation of concentration of PpOplAB and SmPPK2 in CFEs used in the biotransformation of 50 mM **1b** (see main text, Table 6, entry 6 and Figure 3) based on densitometry (integrated density) (see Figure S6).

Entry	Enzyme	Enzyme band area/Total lane area	Content in CFE (%w/w)	[Enzyme] in reactions	TON
1	PpOplAB[a]	6.774/58.106	11.7	0.47 mg/mL (3.4 µM)	4360
2	SmPPK2	7.011/52.618	13.3	0.67 mg/mL (18.1 µM)	785

[a]PpOplA: 3.847 µM (6.6% w/w); PpOplB: 2.927 µM (5.0% w/w), corresponding to a molar ratio of 1.1:1.

5. Procedures for biotransformations

5.1. Biocatalytic reactions using CFEs of PpOplA and PpOplB expressed individually

The pellets of PpOplA and PpOplB were separately resuspended in lysis buffer (ammonium bicarbonate, 50 mM, pH 8.5 containing 4 mM $MgCl_2$), disrupted by sonication with a Branson Sonifier® using following settings: [(30% amplitude, 1 sec pulse on, 4 sec pulse off for 2:30 min) x 2]. Afterwards the centrifugation was done (17000 rpm, 30 min, 4 °C) to remove cell debris. Then the protein concentration (PpOplA and PpOplB) was measured using Bradford assay.
Reactions were performed in 2 mL micro reaction tubes and the samples were shaken horizontally for 24 h at 30 °C and 140 rpm. Two blank reactions were run in parallel in the absence of (i) enzyme and (ii) substrate. After this time, the mixture was filtered via centrifugation and the supernatant was split into two 2 mL micro reaction tubes (each 450 µL). One sample was lyophilized to be used for amino acid derivatization (see section 2.3.1) and further measurements, and the other tube was used for lactam extraction.

5.2. Biocatalytic reactions using lyophilized cells of PpOplA & PpOplB expressed individually

The pellets obtained by autoinduction of PpOplA and PpOplB were lyophilized and tested in the hydrolysis of **1a-c**. Ammonium bicarbonate buffer (50 mM, pH 8.5) containing 4 mM $MgCl_2$ was used for the rehydration of the pellets. The reaction took place in ammonium bicarbonate (50 mM, pH 8.5) containing the lyophilized cells (25 mg/mL for OplA and 55 mg/mL for OplB in 1 mL reaction volume), ATP (5 mM and 12.5 mM final concentration), $MgCl_2$ (4 mM), the substrate (2 mM and 5 mM final concentration). The reaction mixtures and blanks were shaken 48 h (120 rpm, 30 °C, horizontally). Results showed that with using pellets, lower conversions compared to CFE (data not shown). It is worth to mention that when the same pellets in combination with potassium phosphate buffer (50 mM, pH 7.5) as the reaction buffer were used, no conversion was observed in any case (data not shown).

5.3. Biotransformations with CFEs of co-expressed PpOplAB

Biotransformations were performed on an analytical scale (500 µL if not stated otherwise) in 1.5 mL micro reaction tubes that were incubated in an Eppendorf Thermomixer at 900 rpm. All components were combined, as indicated in the table footnotes. 25 µL reaction volume were used for analysis by LC-MS (see section 9.2 for details).

6. Influence of [NH_4HCO_3] on product formation in the conversion of 1b by CFE of PpOplAB

Table S2. Influence of [NH_4HCO_3] on product formation.[a]

Entry	NH_4HCO_3 [mM]	**2b** [mM]
1	5	2.9 ± 0.2
2	10	4.0 ± 0.1
3	15	4.4 ± 0.1
4	30	6.3 ± 1.0
5	55	6.5 ± 0.3

[a]Reaction conditions: 2 mg/mL PpOplAB CFE (total protein content), 10 mM **1b**, 12.5 mM ATP, HEPES buffer (50 mM, pH 8.0, 6.8–7.7 mM $MgCl_2$), 30 °C, 900 rpm, 20 h. NH_4HCO_3 was added from a stock solution (500 mM in H_2O) to reach final concentration of 10, 15, 30 and 55 mM. Mean of triplicates with standard deviation.

7. Time study of the conversion of 1b with CFE of PpOplAB

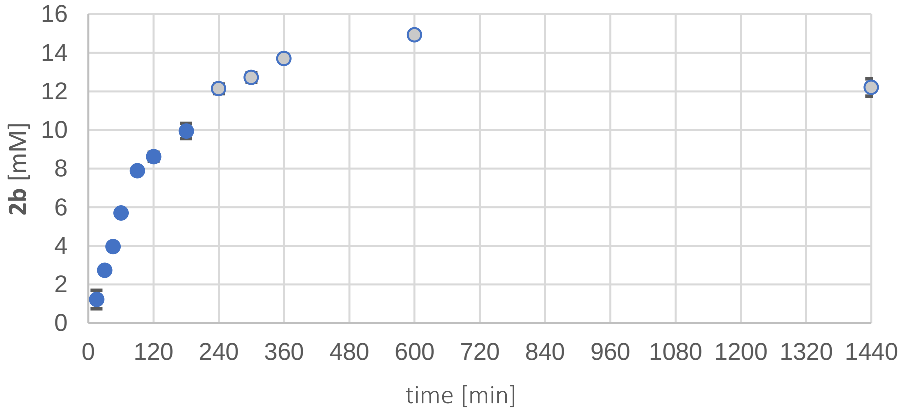

Figure S7. Time course of the PpOplAB-catalyzed hydrolysis of 25 mM of **1b** with ATP cofactor regeneration based on SmPPK2 (0.5 mM ATP). The assay (1 mL volume) was performed in triplicates and 25 µL sample were taken after 15, 30, 45, 60, 120, 180, 240, 360, 600, and 1440 min. Grey dots represent data out of calibration range (0.5–10 mM), which were then obtained by dilution to match the calibration curve (15 µL sample + 20 µL H_2O).

8. Synthesis of reference amino acids

2a-c were purchased from Sigma Aldrich

Synthesis of 2d-g as hydrochloride salts

100 mg of **1d-g** were dissolved under stirring in 1 mL concentrated HCl in a microwave reaction tube (13 mL). The tube was sealed and the mixture heated at 80 °C for 5 days. After completion of the reaction (no lactam present, as monitored by LC-MS), the reaction mixture was cooled down to room temperature and concentrated using a speed vac and dried at 80 °C in the oven overnight. The obtained salts or oily substances still contained water. The water content was determined by ^{1}H-NMR by using acetic acid as internal standard (2 µL, 2 mg, 33.3 µmol, added to 700 µL sample in D_2O, Table S3). The purity of the samples was taken into account for the preparation of the calibration curves.

Table S3. Mass of amino acid hydrochlorides added to NMR samples and calculated water content.

Amino acid hydrochloride salt	Mass added (mg)	Water content (% w/w)
**2d**	8.0	9.4
**2e**	8.8	13.1
**2f**	11.7	18.2
**2g**	11.2	18.3

4-carboxypentan-1-aminium chloride (**2d**): ^{1}H NMR (300 MHz, D_2O) δ 2.90 – 2.86 (m, 2H), 2.46 – 2.39 (m, 1H), 1.59 – 1.51 (m, 2H), 1.42 – 1.39 (m, 2H), 1.03 (d, 3H). ^{13}C NMR (75 MHz, D_2O) δ 181.2, 39.3, 38.7, 29.6, 24.4, 16.3.
4-carboxy-3-methylbutan-1-aminium chloride (**2e**): ^{1}H NMR (300 MHz, D_2O) δ 2.98 – 2.91 (m, 2H), 2.35 – 2.15 (m, 2H), 1.95 – 1.88 (m, 1H), 1.67 – 1.44 (m, 2H), 0.88 (d, 3H). ^{13}C NMR (75 MHz, D_2O) δ 177.6, 40.7, 37.5, 33.2, 27.3, 18.3.[2]
4-carboxy-2-methylbutan-1-aminium chloride (**2f**): ^{1}H NMR (300 MHz, D_2O) δ 2.92 – 2.71 (m, 2H), 2.46 – 2.27 (m, 2H), 1.81 – 1.60 (m, 2H), 1.48 – 1.38 (m, 1H), 0.90 (d, 3H). ^{13}C NMR (75 MHz, D_2O) δ 178.3, 44.7, 30.8, 30.5, 28.1, 15.7.
5-carboxypentan-2-aminium chloride (**2g**): ^{1}H NMR (300 MHz, D_2O) δ 3.29 – 3.23 (m, 1H), 2.34 – 2.30 (m, 2H), 1.61 – 1.46 (m, 4H), 1.18 (d, 3H). ^{13}C NMR (75 MHz, D_2O) δ 178.0, 47.5, 33.2, 33.1, 20.1, 17.5.

9. Analytical methods

9.1. Gas chromatography & mass spectrometry

9.1.1. Derivatization

The biotransformation samples were filtered and dried by lyophilization in 2 mL micro reaction tubes. After lyophilization, the resulting residue was taken up in 700 µL MeOH containing 5% DMAP, and 150 µL ethyl chloroformate was added. Then samples were incubated at 50 °C (horizontal shaking, closed vials, 650 rpm). After 0.5 h, 75 µL ethyl chloroformate was added to each tube and the tubes were shaken for another 0.5 h (vertical shaking, open vials, 550 rpm). Afterwards, the solvent was removed under air flow and 700 µL of 2% aq. HCl solution was added. The extraction was done with ethyl acetate (4 x 400 µL) containing 5 mM behenic acid methyl ester as internal standard. The combined organic phases were dried using anhydrous Na_2SO_4 and then air flow till complete dryness. Then the residues were redissolved in 150 µL ethyl acetate and transferred to plastic GC-vials and measured by GC-FID and GC-MS.
GC-MS: 7890A GC System (Agilent Technologies, Santa Clara, CA, USA), equipped with a 5975C mass selective detector and an HP-5MS column (5% phenylmethylsiloxane, 30 m x 320 µm x 0.25 µm, J&W Scientific, Agilent Technologies) using He as carrier gas. Injector temperature: 250 °C; Injection volume: 5 µL; Split ratio: 90:1; Flow rate: 0.7 mL/min; Temperature program 1: 100 °C, hold time 0.5 min, 10 °C/min to 300 °C, hold time 0 min; EI mode, energy 70 eV, MS Source: 230 °C, MS Quadrupole: 150 °C. The retention times of different compounds are given in Table S4.

Table S4. The retention times (RT) of lactams and derivatized amino acids analyzed by GC-MS

Compounds	RT [min]
**1a**	3.77
**1b**	4.92
**1c**	5.56
**2a**	7.40
**2b**	8.70
**2c**	9.96

GC-FID: Agilent Technologies 7890 A GC system equipped with an FID-detector and a 7693A Injector in combination with a 7693 Series Autosampler and using a HP-5 column (30 m x 320 µm x 0.25 µm, J&W Scientific, Agilent Technologies) using He as carrier gas. Injector temperature: 250 °C; Injection volume: 5 µL; Split ratio: 90:1; Flow rate: 0.7 mL/min; Temperature program: 100 °C, hold time 0.5 min, 10 °C/min to 300 °C, hold time 0 min. The retention times of different compounds are given in Table S5.

Table S5. The retention times (RT) of lactams and derivatized amino acids analyzed by GC-FID

Compounds	Retention times [min]
**1a**	3.68
**1b**	4.76
**1c**	5.57
**2a**	7.45
**2b**	8.76
**2c**	9.95

9.1.2. Calibration curves for GC-FID

To create the calibration curves, various samples with different concentrations (0.5 mM-10 mM) from 100 mM stock solutions of reference compounds were prepared. For example, for a sample with 2 mM concentration, 20 µL from stock was added into 980 µL potassium phosphate buffer (50 mM, pH 7.5). Each sample was split into two 2 mL micro reaction tubes (each 450 µL). The content of one of the tubes was lyophilized to be used for amino acid derivatization (see 9.1.1.), and the other tube was used for lactam extraction. For this purpose, the samples were first saturated with NaCl then extracted with acetonitrile (4 x 400 µL) containing 2 mM behenic acid methyl ester as internal standard. The combined organic phases were dried using anhydrous Na_2SO_4 and then air flow till complete dryness. Then the residues were redissolved in 150 µL ethyl acetate and transferred to the GC-vials and measured by GC-FID and GC-MS.

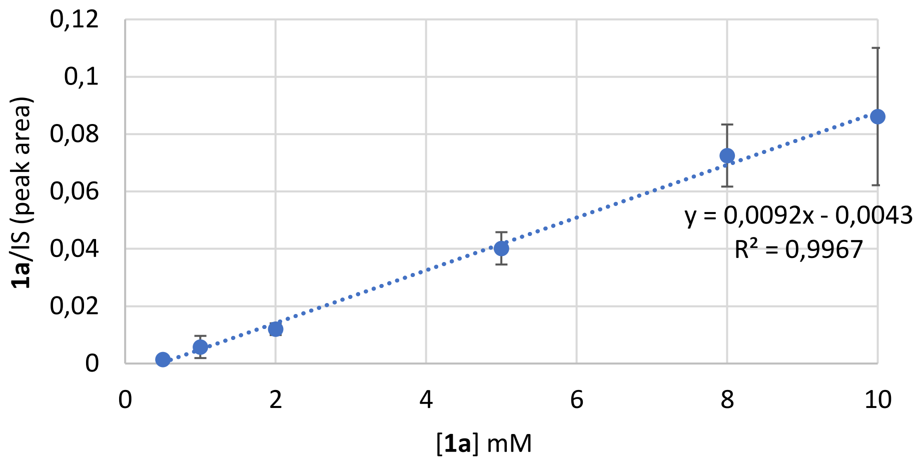

Figure S8. Calibration curve generated for **1a**.

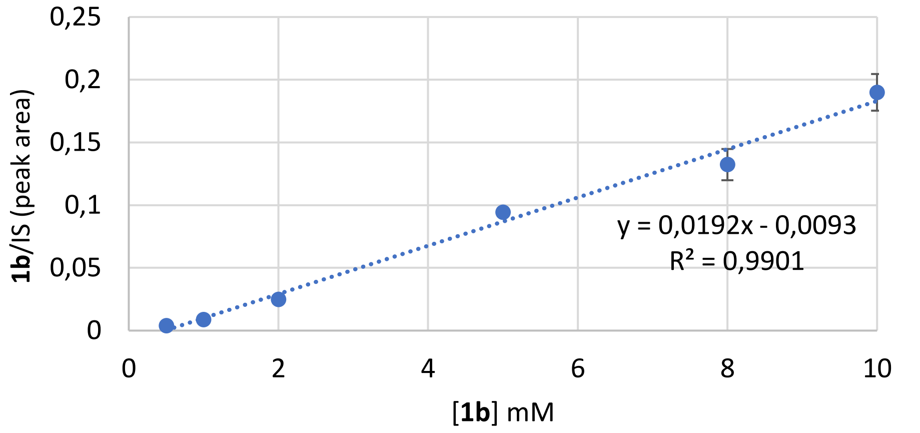

Figure S9. Calibration curve generated for **1b**.

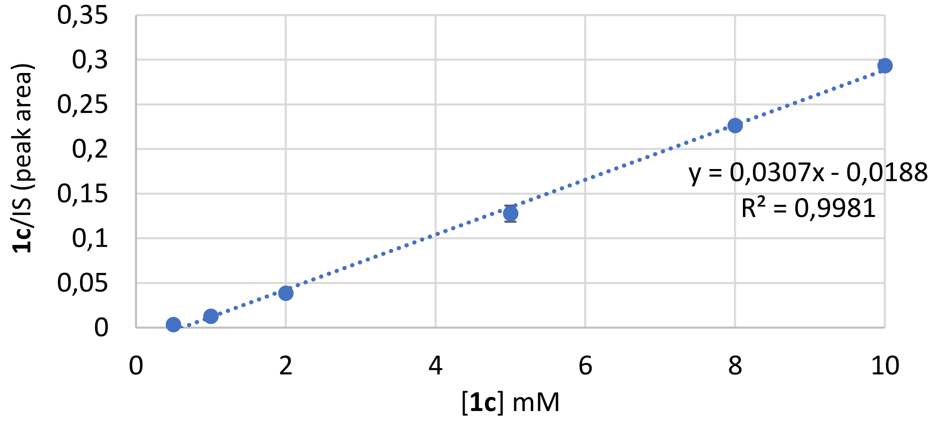

Figure S10. Calibration curve generated for **1c**.

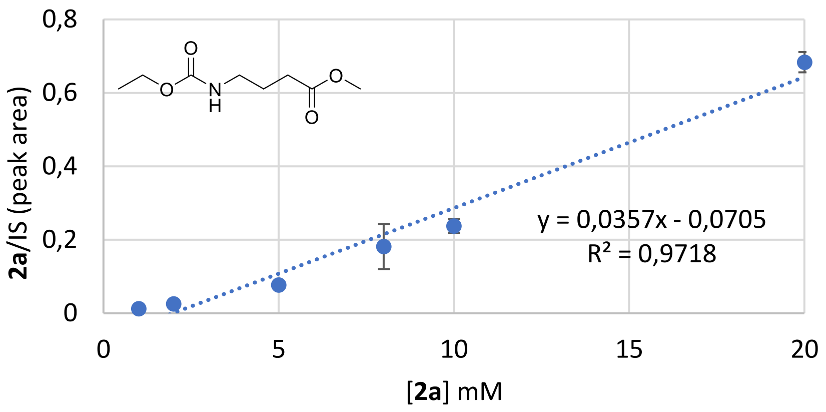

Figure S11. Calibration curve generated for derivatized **2a**.

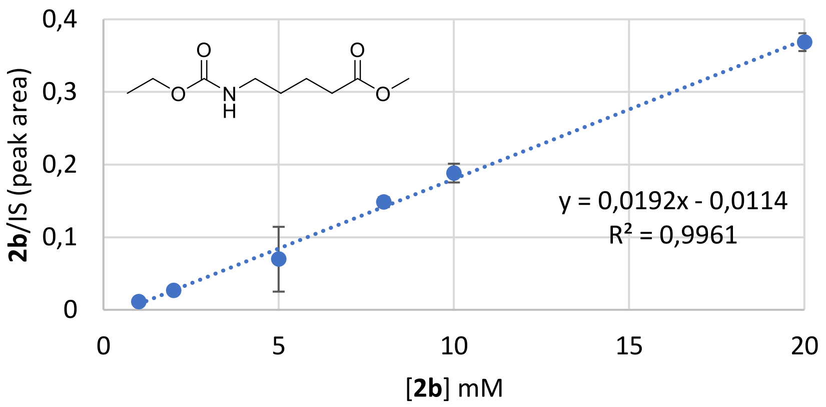

Figure S12. Calibration curve generated for derivatized **2b**.

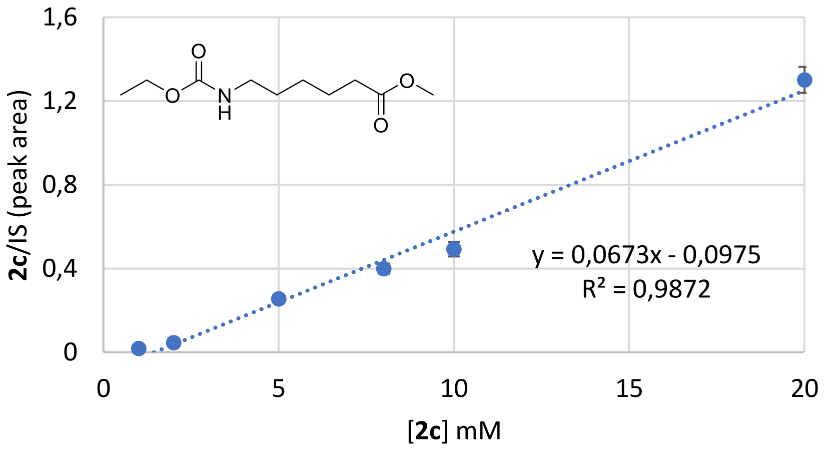

Figure S13. Calibration curve generated for derivatized **2c**.

9.1.3. Representative GC-FID chromatograms

Biotransformations with mixtures of CFEs of PpOplA and PpOplB expressed individually

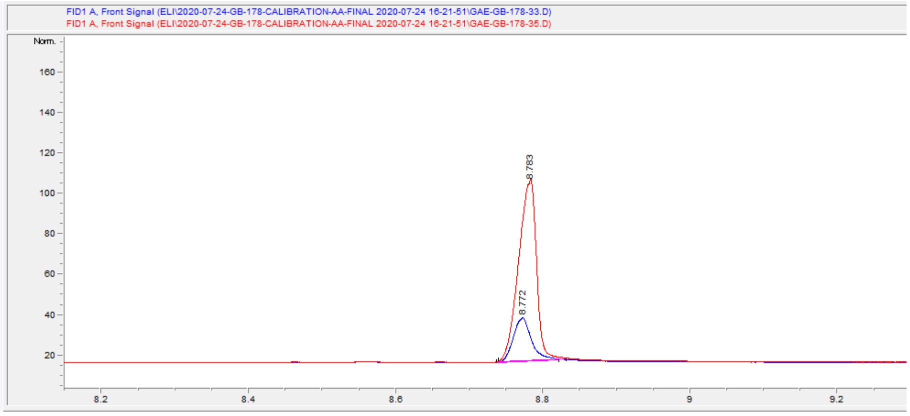

Figure S14. GC trace from reference samples of **2b** used for calibration: 2 mM (blue) and 8 mM (red).

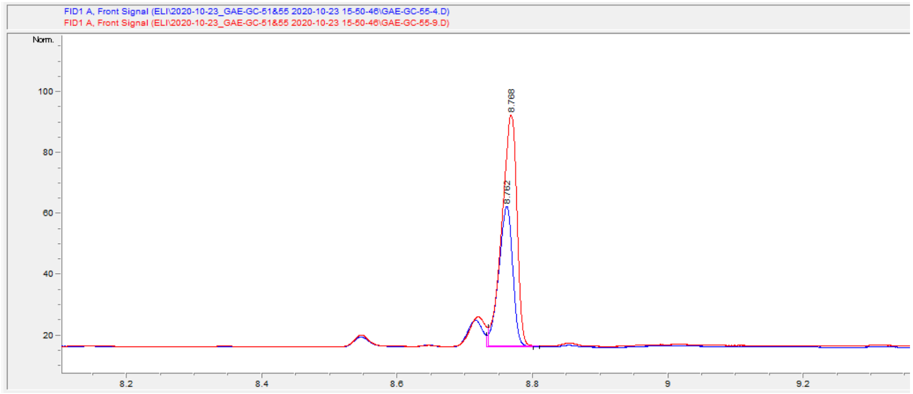

Figure S15. GC trace from the biotransformation of 2 mM **1b** to **2b** (Table 1, entries 2-3) in the presence of 2 mM (blue) and 5 mM (red) ATP. Only **2b** can be seen with that method (8.7 min).

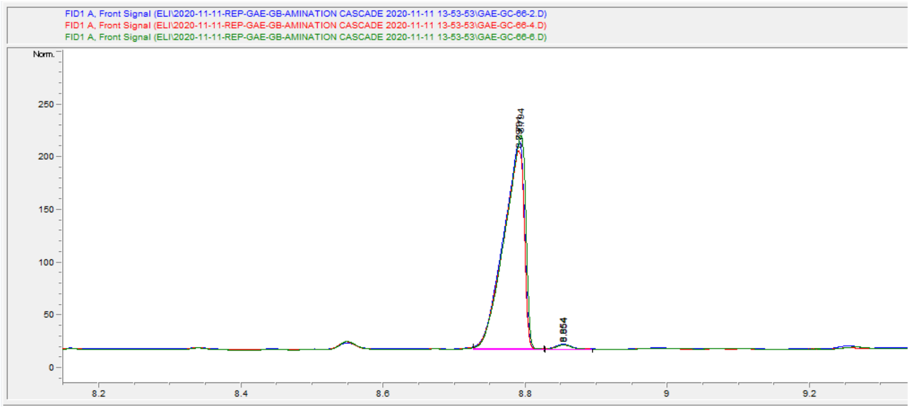

Figure S16. GC trace from biotransformation of **1b** to **2b** (Table 2, entries 1-3) by feeding of ATP (15 mM over 2 h): 6 mM (blue), 8 mM (red) and 10 mM (green) of **1b**. Only **2b** can be seen with that method (8.7 min).

9.2. HPLC-MS

9.2.1. Conditions

HPLC-MS was performed on an Agilent 1260 Infinity HPLC system (G1311B quaternary pump, G1329B autosampler, G1316A thermostatted column compartment, G1314F variable wavelength detector) coupled to an Agilent 6120 single-quadrupole MS detector (settings given in Table S6). The analytes [M+1] were detected by SIM in positive mode and separated using a C18 column (Luna 5 µm, 250 mm × 4.6 mm; Phenomenex) with isocratic elution (0.6 ml/min, ACN+0.1% FA, H_2O+0.1% FA, 80:20) at 30 °C and 1 µL injection volume. Samples used for quantification were prepared by diluting 25 µL biotransformation or calibration mixture with 475 µL acidic workup mix containing L–(+)-α-phenylglycine as internal standard (16% formic acid, 64% acetonitrile, 20% H_2O, 0.1 mM L–(+)-α-phenylglycine, pH ca. 2.5) to ensure reproducible protonation states of the analytes and stable peak areas. The mixture was centrifuged and the supernatant used for analysis (1 µL). A table with all retention times and *m/z* ratios [M+1] is given below (Table S7).

Table S6. MS settings used for lactam and amino acid analysis.

ionization mode	API-ES, positive polarity
drying gas temperature	300 °C
drying gas flow	10.0 L/min
nebulizer pressure	35 psig
capillary voltage	3000 V
signal 1: scan in positive mode	25% cycle time<br>mass range 40–400<br>fragmentor 40 V
signal 2: substrate	25% cycle time<br>fragmentor 40 V<br>SIM see Table S7
signal 3: product	25% cycle time<br>fragmentor 40 V<br>SIM see Table S7
signal 4: internal standard	25% cycle time<br>fragmentor 40 V<br>SIM see Table S7

Table S7. *m/z* values [M+1] and retention time (RT) of lactams and corresponding amino acids.

Lactam	*m/z*, RT	Amino acid	*m/z*, RT
**1a**	86, 4.4 min	**2a**	104, 2.7 min
**1b**	100, 4.4 min	**2b**	118, 2.7 min
**1c**	114, 4.6 min	**2c**	132, 2.7 min
**1d**	114, 4.6 min	**2d**	132, 2.7 min
**1e**	114, 4.6 min	**2e**	132, 2.7 min
**1f**	114, 4.6 min	**2f**	132, 2.8 min
**1g**	114, 4.6 min	**2g**	132, 2.7 min
		L–(+)-α-phenylglycine (IS)	152, 3.5 min

1a **1b** **1c** **1d** **1e** **1f** **1g**

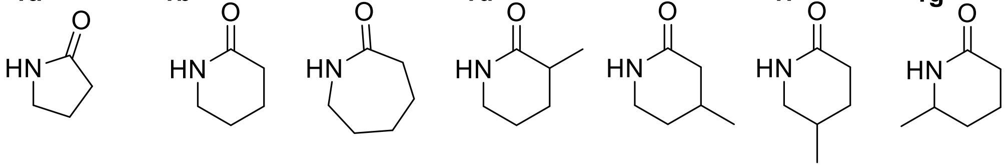

9.2.2. Calibration curves for HPLC-MS

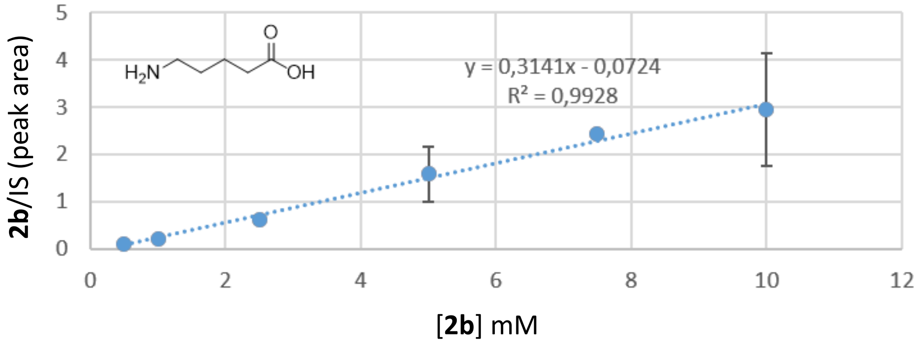

Figure S17. Calibration curve **2b** with reaction background (4 mg/mL PpOplAB (in ammonium bicarbonate buffer 50 mM, pH 8.5, 10 mM $MgCl_2$), 5 mg/mL SmPPK (in HEPES buffer (50 mM, pH 8.0, 10 mM $MgCl_2$, 50 mM NaCl, 5% glycerol), 0.5 mM ATP, 10 mM Graham´s salt, in ammonium bicarbonate buffer (50 mM, pH 8.5, 10 mM $MgCl_2$). Mean of triplicates with standard deviation. Workup: 25 µL sample mixed with 475 µL acidic workup mix (16% formic acid, 64% acetonitrile, 20% H_2O, 0.1 mM L–(+)-α-phenylglycin, pH ca. 2.5).

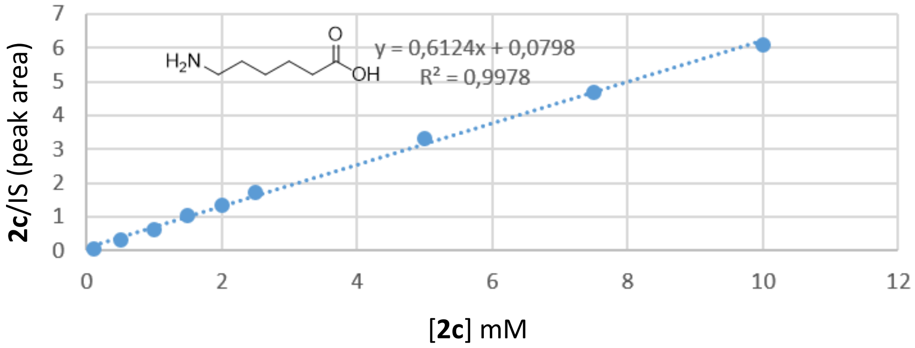

Figure S18. 2c calibration in ammonium bicarbonate buffer (50 mM, pH 8.5, 10 mM $MgCl_2$). Mean of duplicates. Workup: 25 µL sample mixed with 475 µL acidic workup mix (16% formic acid, 64% acetonitrile, 20% H_2O, 0.1 mM L−(+)-α-phenylglycin, pH ca. 2.5)

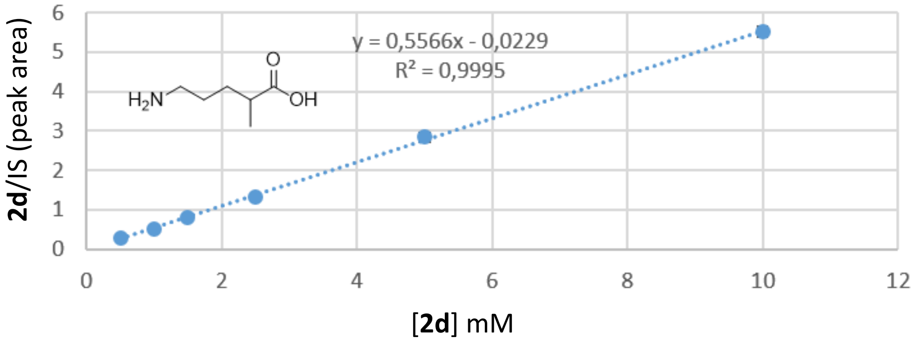

Figure S19. 2d calibration with PpOplAB lysate background (8 mg/mL) in ammonium bicarbonate buffer (50 mM, pH 8.5, 10 mM $MgCl_2$). Mean of triplicates with standard deviation. Workup: 25 µL sample mixed with 475 µL acidic workup mix (16% formic acid, 64% acetonitrile, 20% H_2O, 0.1 mM L−(+)-α-phenylglycin, pH ca. 2.5)

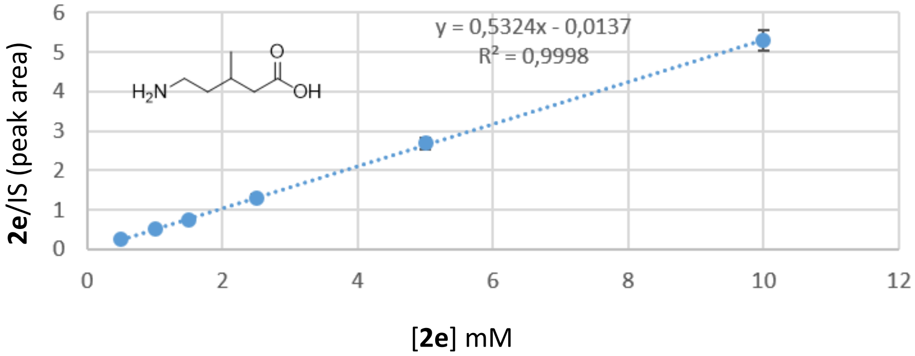

Figure S20. 2e calibration with PpOplAB lysate background (8 mg/mL) in ammonium bicarbonate buffer (50 mM, pH 8.5, 10 mM $MgCl_2$). Mean of triplicates with standard deviation. Workup: 25 µL sample mixed with 475 µL acidic workup mix (16% formic acid, 64% acetonitrile, 20% H_2O, 0.1 mM L−(+)-α-phenylglycin, pH ca. 2.5)

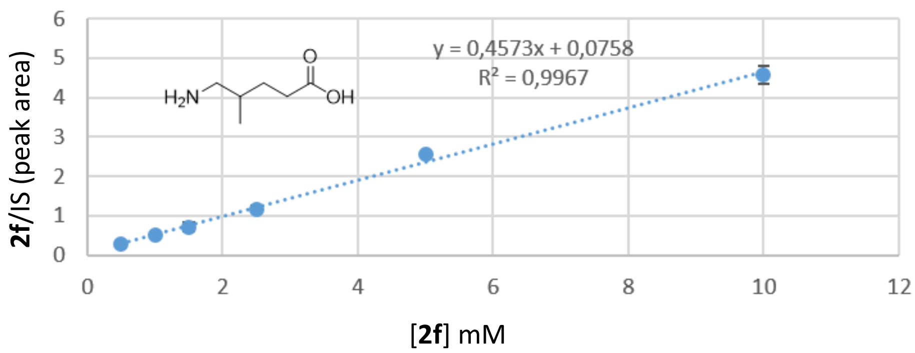

Figure S21. 2f calibration with PpOplAB lysate background (8 mg/mL) in ammonium bicarbonate buffer (50 mM, pH 8.5, 10 mM $MgCl_2$). Mean of triplicates with standard deviation. Workup: 25 µL sample mixed with 475 µL acidic workup mix (16% formic acid, 64% acetonitrile, 20% H_2O, 0.1 mM L−(+)-α-phenylglycin, pH ca. 2.5)

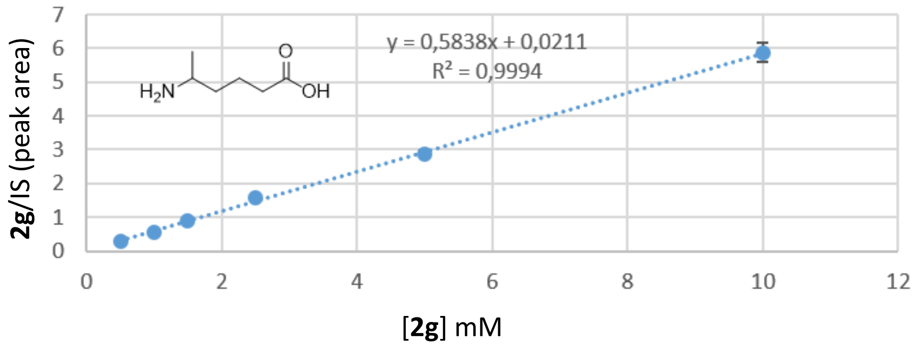

Figure S22. 2g calibration with PpOplAB lysate background (8 mg/mL) in ammonium bicarbonate buffer (50 mM, pH 8.5, 10 mM $MgCl_2$). Mean of triplicates with standard deviation. Workup: 25 µL sample mixed with 475 µL acidic workup mix (16% formic acid, 64% acetonitrile, 20% H_2O, 0.1 mM L−(+)-α-phenylglycin, pH ca. 2.5)

9.2.3. Representative LC-MS chromatograms

Lysate

The lysate of PpOplAB showed no trace of **1a**, **2c**, and only negligeable traces of **2a**, **1b**, **2b**, **1c** and IS (Figure S23).

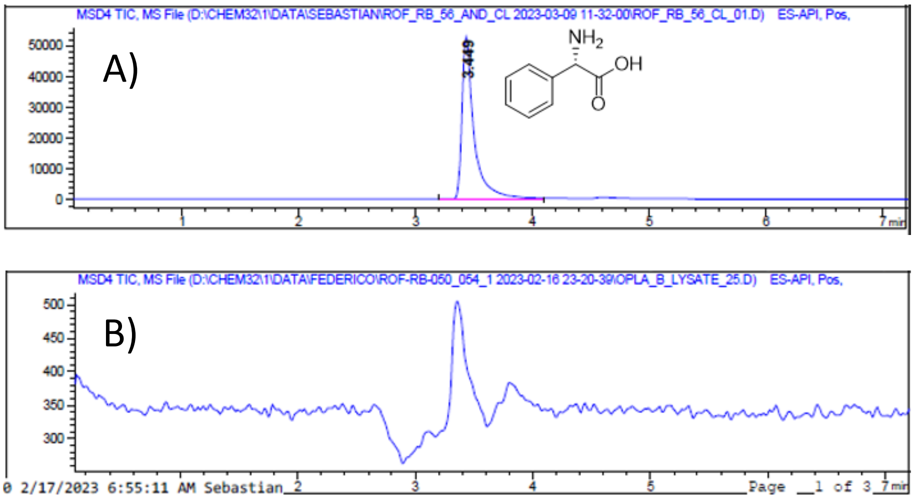

Figure S23. HPLC-MS chromatograms (SIM 152, L−(+)-α-phenylglycine) of A) L−(+)-α-phenylglycine as reference used in the analytical mixture and B) PpOplAB lysate (25 mg/mL) in ammonium bicarbonate buffer (50 mM, pH 8.5, 10 mM $MgCl_2$).

References

2b

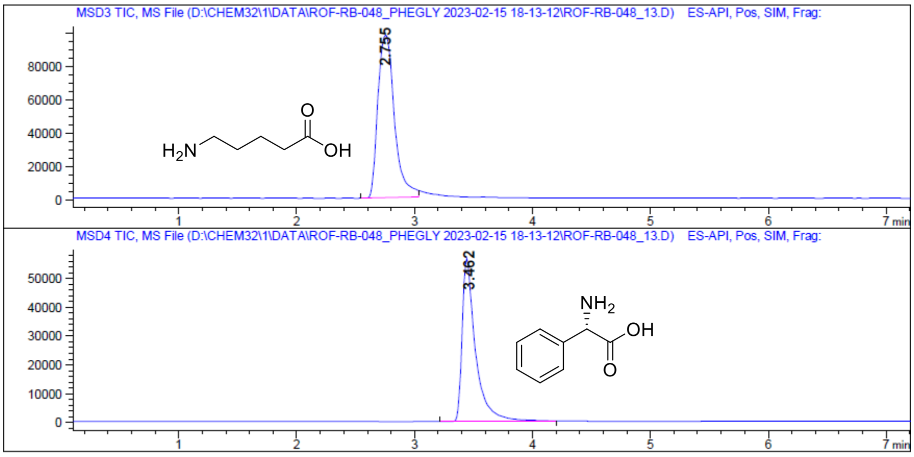

Figure S24. HPLC-MS chromatograms (SIM) from reference **2b** (5 mM) in ammonium bicarbonate buffer (50 mM, pH 8.5, 10 mM $MgCl_2$): upper SIM 118 for **2b** and lower SIM 152 for IS.

2c

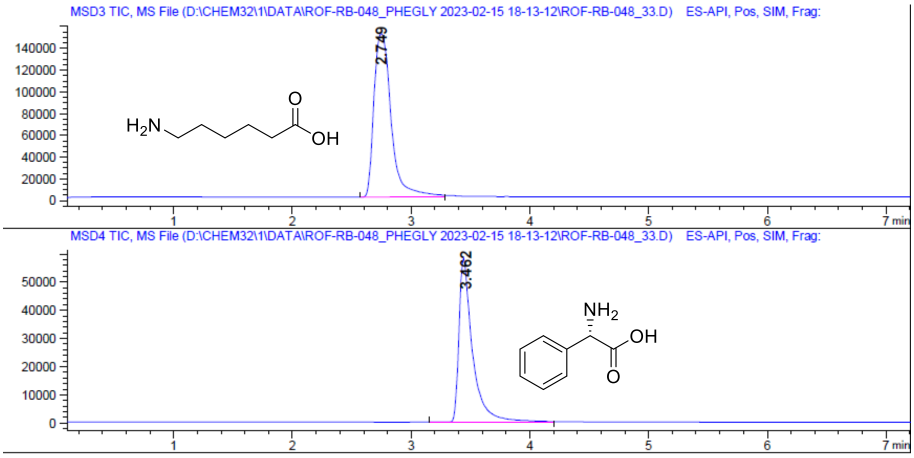

Figure S25. HPLC-MS chromatograms (SIM) from reference **2c** (5 mM) in ammonium bicarbonate buffer (50 mM, pH 8.5, 10 mM $MgCl_2$): upper SIM 132 for **2c** and lower SIM 152 for IS.

Biotransformations with PpOplAB

1a to **2a**

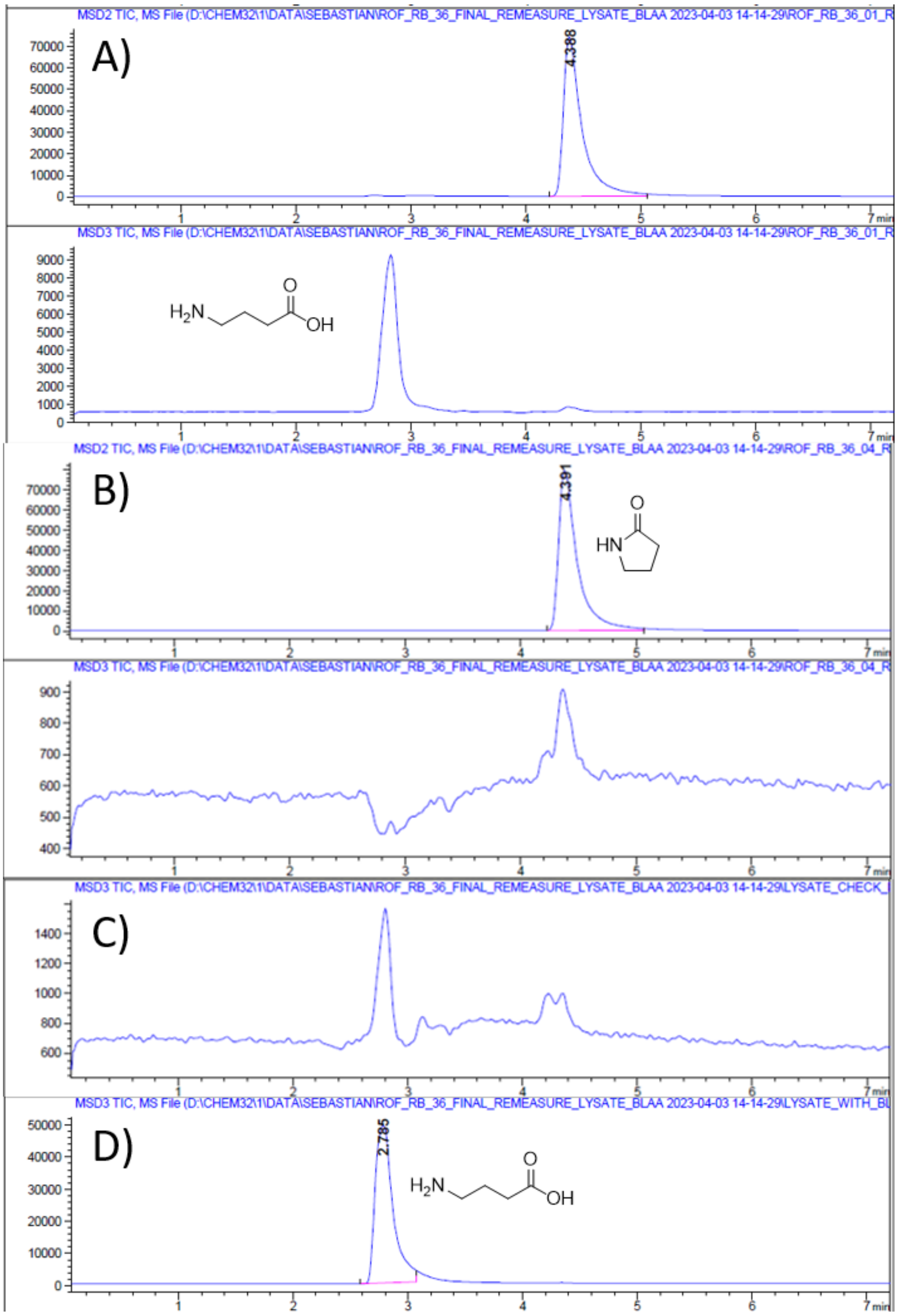

Figure S26. HPLC-MS chromatograms (SIM) from A) biotransformation of 10 mM **1a** with 8 mg/mL PpOplAB CFE in ammonium bicarbonate buffer (50 mM, pH 8.5, 10 mM $MgCl_2$): upper SIM 86 for **1a** and lower SIM 104 for **2a**, corresponds to data from Table 3, entry 1; B) 10 mM **1a** in ammonium bicarbonate buffer (50 mM, pH 8.5, 10 mM $MgCl_2$): upper SIM 86 for **1a** and lower SIM 104 for **2a**; C) 8 mg/mL PpOplAB lysate: SIM 104 for **2a**; D) 5 mM **2a** spiked with 8 mg/mL lysate: SIM 104 for **2a**.

1b to **2b**

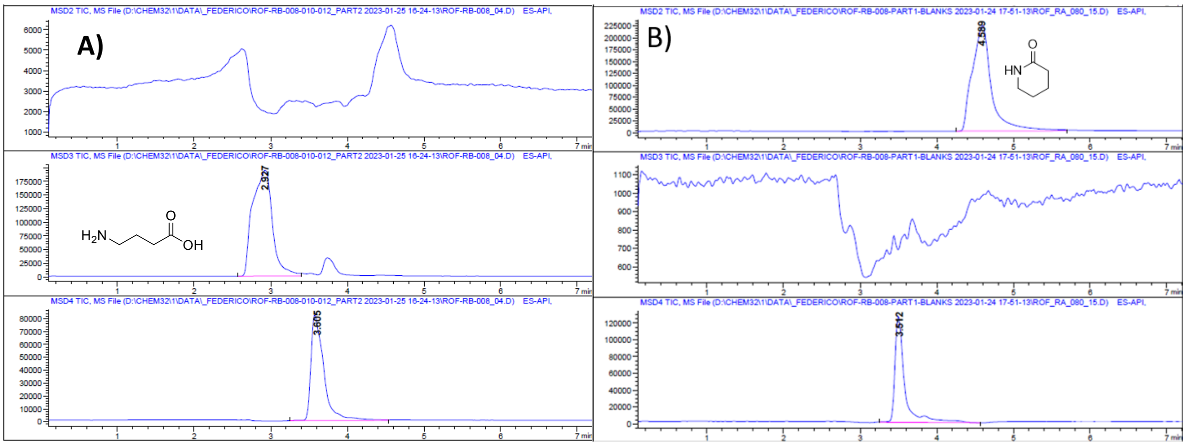

Figure S27. HPLC-MS chromatograms (SIM) from A) biotransformation of 10 mM **1b** with 4 mg/mL PpOplAB CFE in ammonium bicarbonate buffer (50 mM, pH 8.5, 10 mM $MgCl_2$): upper SIM 100 for **1b**, middle SIM 118 for **2b**, lower SIM 152 for IS and B) 10 mM **1b** in ammonium bicarbonate buffer (50 mM, pH 8.5, 10 mM $MgCl_2$), control reaction without enzyme: upper SIM 100 for **1b**, middle SIM 118 for **2b** and lower SIM 152 for IS.

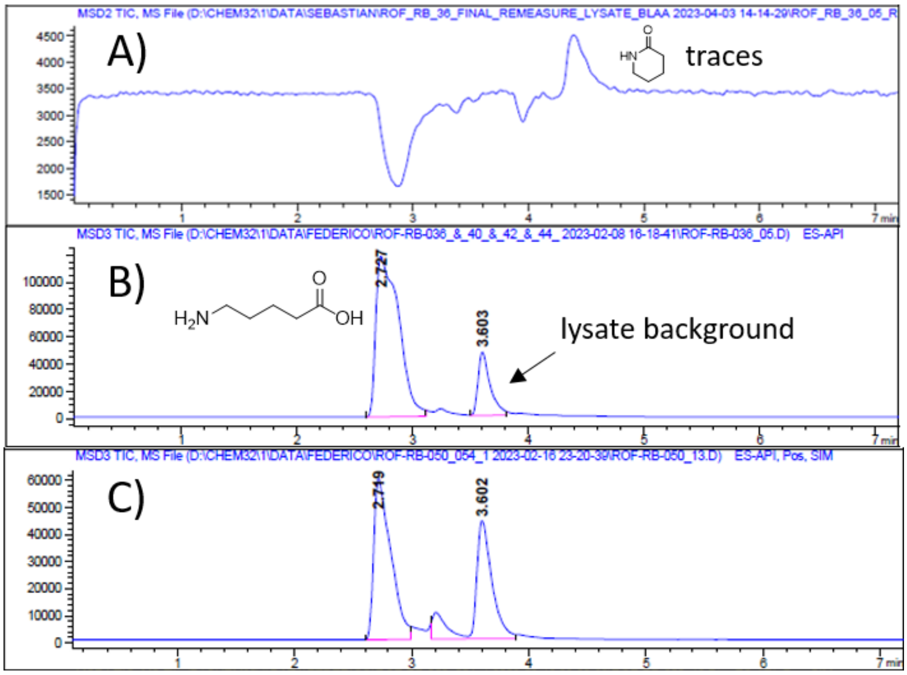

Figure S28. HPLC-MS chromatograms (SIM) from biotransformation of 10 mM **1b** with 8 mg/mL PpOplAB CFE in ammonium bicarbonate buffer (50 mM, pH 8.5, 10 mM $MgCl_2$): A) SIM 100 for **1b** and B) SIM 118 for **2b**, corresponds to data from Table 3, entry 2; C) calibration sample of 5 mM **2b** with reaction background (CFE): SIM 118 for **2b**.

1c to **2c**

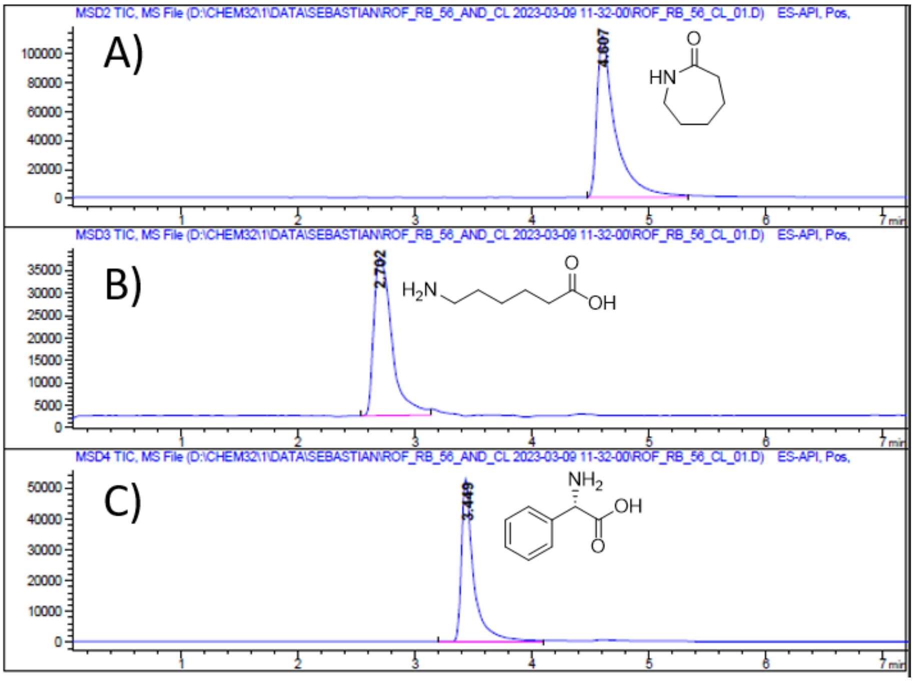

Figure S29. HPLC-MS chromatograms (SIM) from biotransformation of 10 mM **1c** with 8 mg/mL PpOplAB CFE in ammonium bicarbonate buffer (50 mM, pH 8.5, 10 mM $MgCl_2$): A) SIM 114 for **1c**, B) SIM 132 for **2c**, C) SIM 152 for IS. Corresponds to data from Table 3, entry 3.

1d to **2d**

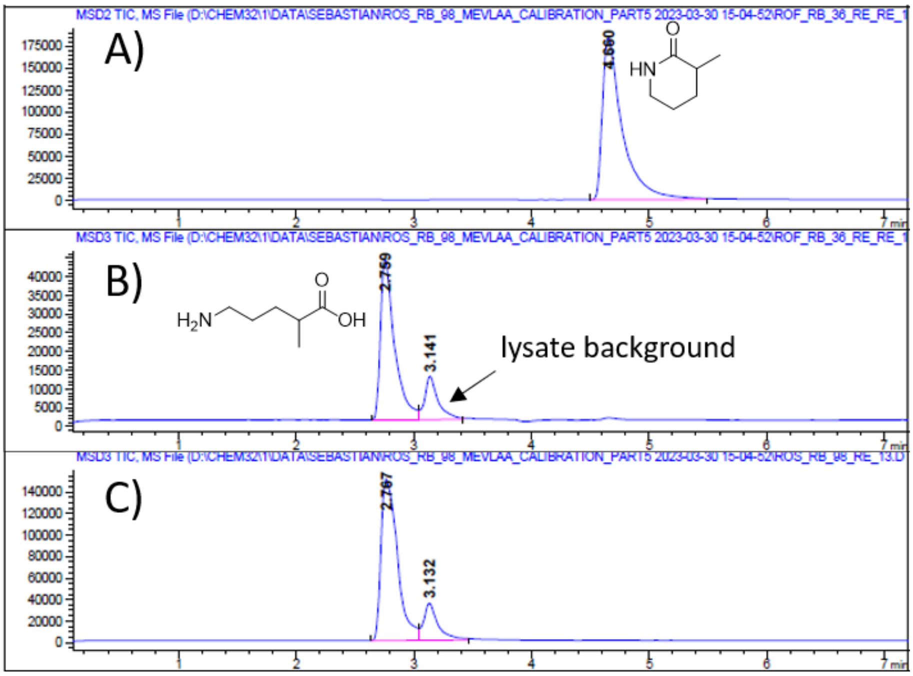

Figure S30. HPLC-MS chromatograms (SIM) from biotransformation of 10 mM **1d** with 8 mg/mL PpOplAB CFE in ammonium bicarbonate buffer (50 mM, pH 8.5, 10 mM $MgCl_2$): A) SIM 114 for **1d**, B) SIM 132 for **2d**, corresponds to data from Table 3, entry 4; C) calibration sample of 5 mM **2d** with reaction background (CFE): SIM 132 for **2d**.

1e to **2e**

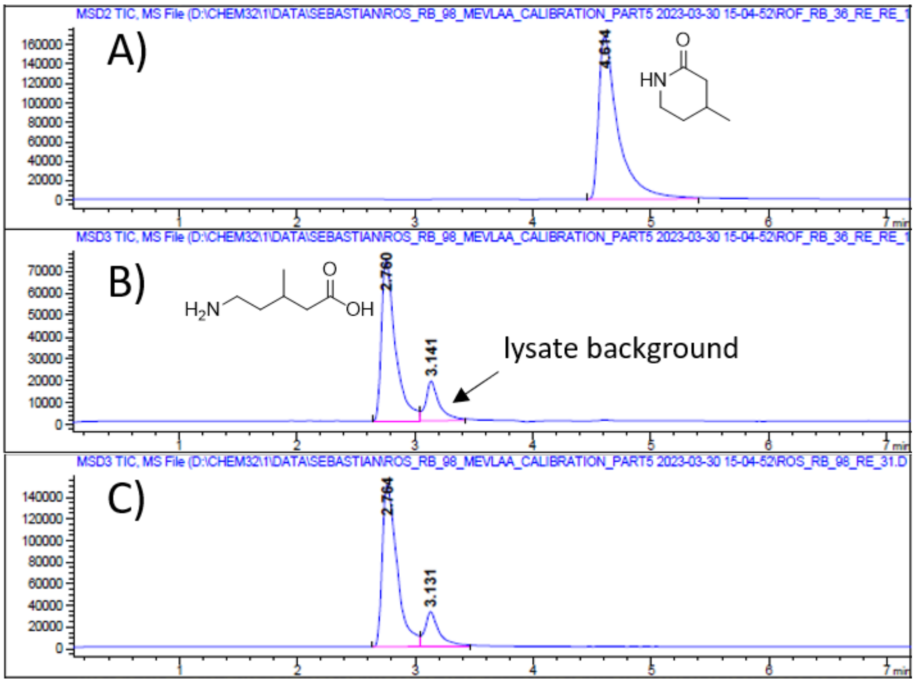

Figure S31. HPLC-MS chromatograms (SIM) from biotransformation of 10 mM **1e** with 8 mg/mL PpOplAB CFE in ammonium bicarbonate buffer (50 mM, pH 8.5, 10 mM $MgCl_2$): A) SIM 114 for **1e**, B) SIM 132 for **2e**, corresponds to data from Table 3, entry 5; C) calibration sample of 5 mM **2e** with reaction background (CFE): SIM 132 for **2e**.

1f to **2f**

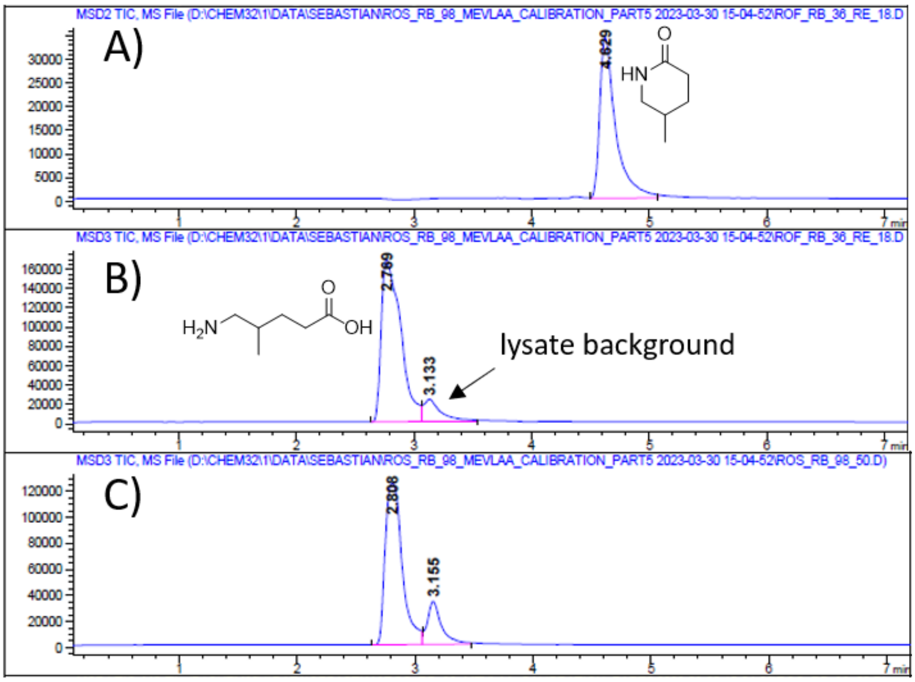

Figure S32. HPLC-MS chromatograms (SIM) from biotransformation of 10 mM **1f** with 8 mg/mL PpOplAB CFE in ammonium bicarbonate buffer (50 mM, pH 8.5, 10 mM $MgCl_2$): A) SIM 114 for **1f**, B) SIM 132 for **2f**, corresponds to data from Table 3, entry 6; C) calibration sample of 5 mM **2f** with reaction background (CFE): SIM 132 for **2f**.

1g to **2g**

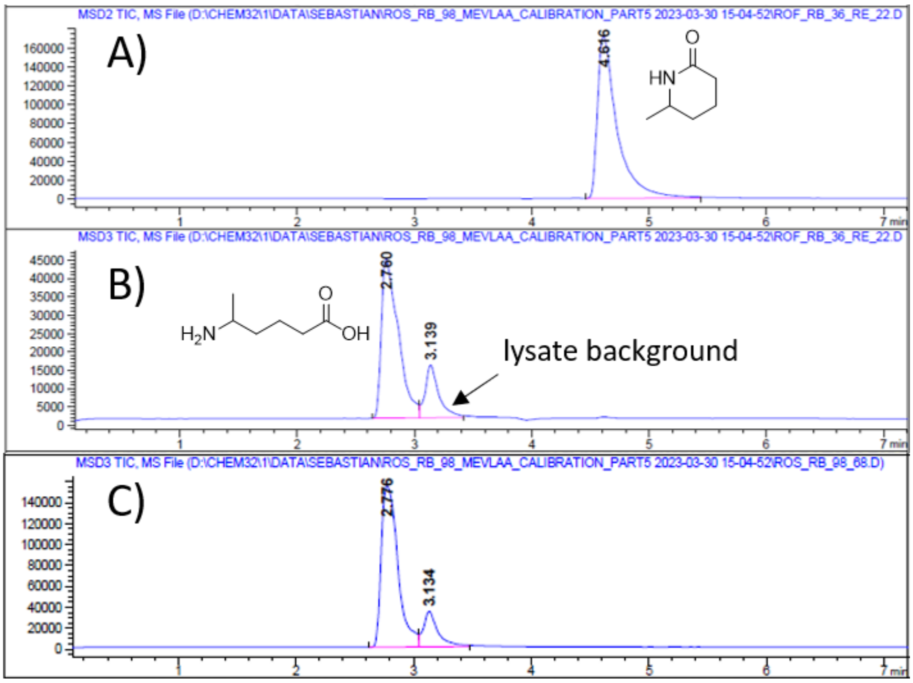

Figure S33. HPLC-MS chromatograms (SIM) from biotransformation of 10 mM **1g** with 8 mg/mL PpOplAB CFE in ammonium bicarbonate buffer (50 mM, pH 8.5, 10 mM $MgCl_2$): A) SIM 114 for **1g**, B) SIM 132 for **2g**, corresponds to data from Table 3, entry 7; C) calibration sample of 5 mM **2g** with reaction background (CFE): SIM 132 for **2g**.

Biotransformation of **1b** to **2b** by purified PpOplAB

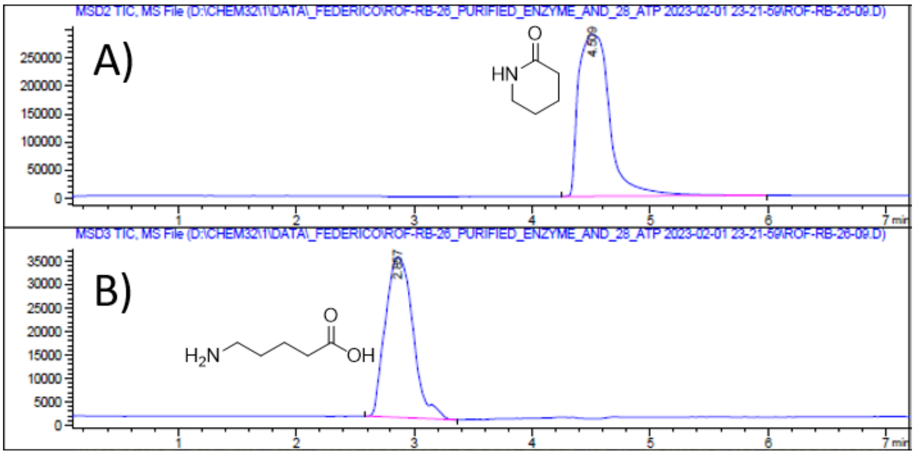

Figure S34. HPLC-MS chromatograms (SIM) from biotransformation of 10 mM **1b** with 1 mg/mL purified PpOplAB in ammonium bicarbonate buffer (50 mM, pH 8.5, 10 mM $MgCl_2$): A) SIM 100 for **1b**, B) SIM 1118 for **2b**.

Biotransformations of **1b** by PpOplAB with phosphodonors **3a-c** compared to with ATP

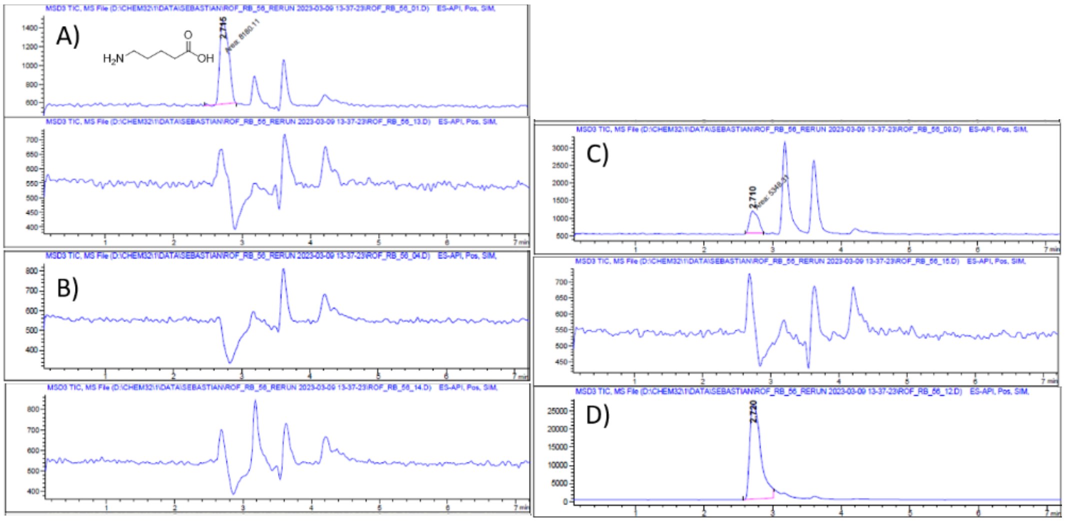

Figure S35. HPLC-MS chromatograms (SIM 118 for **2b**) of A) upper: biotransformation with carbamoyl phosphate (**3a**) and lower: corresponding control reaction without CFE; B) upper: biotransfromation with phosphoenolpyruvic acid (**3c**) and lower: corresponding control reaction without CFE; C) upper: biotransformation with acetyl phosphate (**3b**) and lower: corresponding control reaction without CFE; and D) biotransformation with ATP (2.9 mM **2b** formed). RT from **2b**: 2.7 min. Corresponds to data from Table 7.

ATP-regeneration with SmPPK2

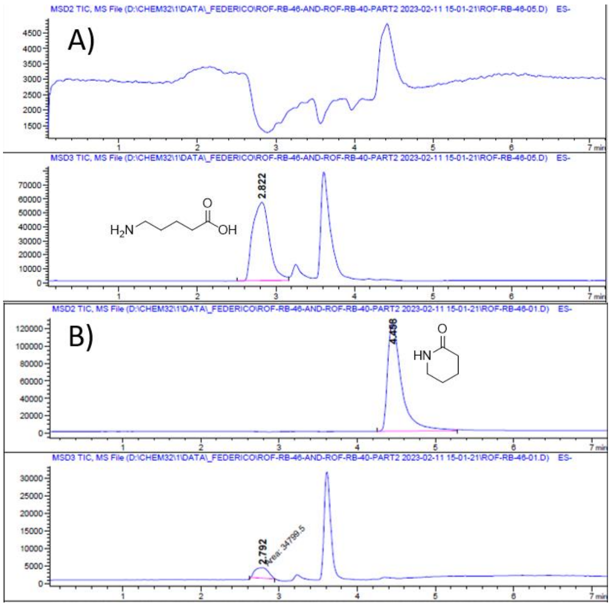

Figure S36. HPLC-MS chromatograms with SIM 100 for **1b**, upper trace, and SIM 118 for **2b**, lower trace of A) reaction with SmPPK2 (>99% conversion, 100% relative activity based on [**2b**]) and B) reaction without SmPPK2 (3% relative activity). Corresponds to data from Table 5.

10. Analysis of enantioselectivity

The synthesis of the reference amino acid *rac*-**2e** was performed as indicated section 8, p. 10. After 8 days, the reaction was cooled down, and then the pH was brought to 6-7 using NaOH before drying the sample using a speedvac (vacuum concentrator).

Derivatization of 2e

The reference amino acid *rac*-**2e** was derivatized to *rac*-5-(((benzyloxy)carbonyl)amino)-3-methylpentanoic acid for analysis on chiral phase HPLC. The synthesis was adapted from the literature.[3]

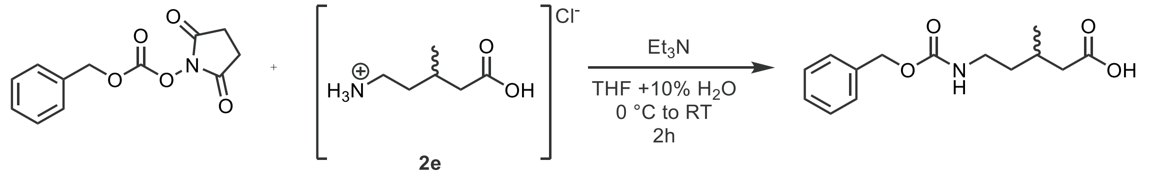

150 mg of *N*-(Benzyloxycarbonyloxy)succinimide (0.89 mmol, 1 equivalent) was dissolved in 4.47 mL of THF with 10 vol% water (447 µL), employing magnetic stirring. Subsequently, triethylamine (Et_3N) (187.2 µL, 1.34 mmol, 1.5 equivalents) was added, and the reaction mixture was kept at 0 °C with an ice bath for 10 minutes. Following this, 223 mg (0.89 mmol, 1 equivalent) of **2e** as ammonium chloride salt was introduced in one portion, and after an additional 10 minutes of stirring, the ice bath was removed, allowing the reaction to reach room temperature. Confirmation of reaction completion was made after 2 hours by HPLC-MS analysis using the method of an isocratic gradient 80:20 ACN (+0.1% formic acid) and water (+0.1% formic acid) at a ratio of 80:20 for 8 minutes. The reaction mixture was acidified with 2 vol% HCl until the pH reached below 3, followed by four extractions with 15 mL of ethyl acetate. The collected organic phases were then dried using sodium sulfate (Na_2SO_4), and the solvent was evaporated under reduced pressure, resulting in 510.9 mg of crude product. The reaction crude was purified by solubilization in 2 mL of acetonitrile and subsequent injection into the preparative HPLC system. The system employed a gradient elution procedure featuring a Phenomenex Luna® 5 µm C18(2) 100 Å column sized at 21.2 mm x 250 mm (00G-4252-P0-AX), with a flow rate of 30 mL/min. The purification method followed these conditions: Initially, a gradient of 60:40 water (with 0.1% TFA) to ACN (with 0.1% TFA) was applied for 2 minutes. Over 35 minutes, the gradient transitioned to a composition of 40:60 water (with 0.1% TFA) to ACN (with 0.1% TFA). Subsequently, after 40 minutes, the composition was adjusted to 100% ACN and kept for 5 minutes. The collected fractions were dried under reduced pressure and subjected to analysis, isolating the desired product as a white solid weighing 146.7 mg (62.2 % yield).

NMR *rac*-5-(((benzyloxy)carbonyl)amino)-3-methylpentanoic acid (in agreement with the literature,[2] see Figure S46)

^{1}H NMR (300 MHz, $CDCl_3$) δ 7.41 – 7.29 (m, 5H, $H_{arom.}$), 5.10 (s, 2H, -O-C$\underline{H}_2$-Ar), 3.23 (s, 2H, -CH-CH_2-C$\underline{H}_2$-NH-), 2.42 – 2.26 (m, *J* = 15.2, 5.8 Hz, 1H, part A of HOOC-C$\underline{H}_2$-CH-), 2.28 – 2.14 (m, *J* = 15.3, 7.3 Hz, 1H, part B of HOOC-C$\underline{H}_2$-CH-), 2.12 – 1.92 (m, *J* = 13.5, 6.7 Hz, 1H, HOOC-CH_2-C$\underline{H}$-CH_2-), 1.66 – 1.50 (m, *J* = 13.3, 7.4 Hz, 1H, part A of -CH-C$\underline{H}_2$- CH_2-NH), 1.49 – 1.33 (m, 1H, part B of -CH-C$\underline{H}_2$- CH_2-NH), 1.00 (d, *J* = 6.3 Hz, 3H, -C$\underline{H}_3$).

^{13}C NMR (75 MHz, $CDCl_3$) δ 178.33 (-$\underline{C}$OOH), 156.64 (-NH-$\underline{C}$=O-O-), 136.56 ($Cquat_{arom}$), 128.65 (C_{arom}), 128.26 (C_{arom}), 128.21 (C_{arom}), 66.91 (-NH-C=O-O-CH_2-Ar), 41.31 (HOOC-$\underline{C}H_2$-CH), 39.00 (-CH-CH_2-$\underline{C}H_2$-NH-), 36.55 (-CH-$\underline{C}H_2$-CH_2-NH-), 27.61 (-$\underline{C}$H-CH3), 19.72(-CH_3).

Analysis of the biotransformations

The biotransformation of **1e** was performed as indicated in the substrate screening, Table 3 (only difference: 8 mg/mL PpOplAB CFE). After incubation of the reaction, the samples were basified by adding 50 µL of a 10 M NaOH solution in water. The pH was checked, and an additional 50 µL base was added as needed to ensure the pH was above 10.

For the analysis of **1e**, the substrate was extracted from the biotransformation mixture using ethyl acetate: 200 µL of EtOAc was added to the samples, followed by centrifugation at maximum speed for 10 minutes. From the upper organic phase, 150 µL was carefully extracted. This extraction process was repeated three times, but in the last repetition, only 100 µL of EtOAc was added. The combined organic phases were then dried with sodium sulfate (Na_2SO_4). Finally, 150 µL of the extracted material was transferred into a plastic GC vial and analyzed.
The determination of the enantiomeric excess of **1e** was conducted as reported in the literature[4] using Gas Chromatography with Flame Ionization Detection (GC-FID) and the following conditions: HYDRODEX β-6TBDM column, 50 m, 0.25 mm ID, carrier gas: H_2, flow rate 1 mL/min, injection volume 2 µL with a Split/Splitless ratio of 30:1, injection temperature 230°C; oven temperature 50°C, heating rate: 5°C/min to 80 °C, then 0.5 °C/min to 170 °C, and 10 °C to 200 °C. Enantiomer elution based on the literature[4]: (*S*)-**1e**: 140.3 min, (*R*)-**1e**: 141.5 min.

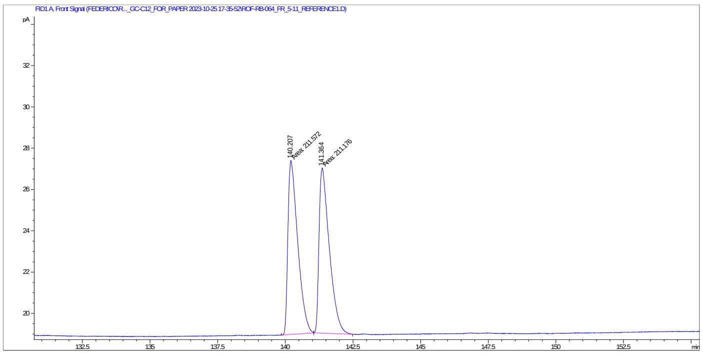

Figure S37. Trace obtained from *rac*-**1e** on chiral phase GC.

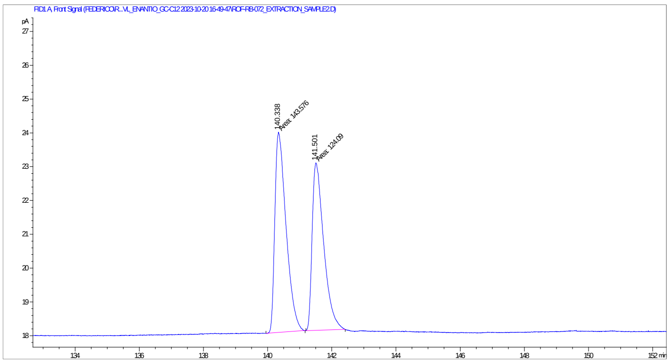

Figure S38. Trace obtained from the biotransformation of *rac*-**1e** on chiral phase GC indicating enrichment of (*S*)-**1e** with 8% ee.

For the analysis of **2e**, to the remaining aqueous phase, 150 µL of a 150 mM stock solution *N*-(benzyloxycarbonyloxy)succinimide in acetonitrile (ACN) was added.[5] The reaction was then allowed to react for 1.5 h in a Benchtop thermomixer at 50 °C and 600 rpm. Following the reaction, the Eppendorf tubes were allowed to cool down to room temperature. Subsequently, 700 µL of a 4 M HCl

solution in water was added, and the pH was checked and adjusted to be less than 3 by adding more acid if necessary. The mixture was brought to dryness using a SpeedVac. The whitish solid, with a few brownish residues, was resuspended in 200 µL of ACN. The resuspension was vortexed, and sometimes, a pipette tip was used to ensure complete dissolution. The mixture was then incubated at 50 °C and 600 rpm for 10 min. The mixture was centrifuged at maximum speed for 10 min. After centrifugation, 150 µL of the supernatant was transferred into a plastic GC vial.

The presence of the desired peak (264 m/z using the negative ionization method) was confirmed using High-Performance Liquid Chromatography-Mass Spectrometry (HPLC-MS) analysis with the Luna(R) 5µm C18(2) 100 Å column (250 x 4.6 mm). The analysis was conducted using an isocratic elution method with a ratio of 80:20 ACN (+ 0.1% Formic Acid) to water (+ 0.1% Formic Acid) for 8 minutes. The analysis was carried out at a constant temperature of 30 °C, with a flow rate set at 0.6 mL/min, and samples were injected using a 5 µL volume. Comparison with elution of the authentic reference material confirmed that derivatized **2e** was present.

The enantiomeric excess analysis of the product was conducted using a Shimadzu HPLC-DAD (High-Performance Liquid Chromatography with Diode Array Detection) employing a Chiralpak column IA (from DAICEL) with dimensions of 4.6 mm ID and 250 mm L with a particle size of 5 µm (part no. 80325). The analysis was performed in isocratic elution technique using a heptane:2-propanol ratio of 94:6. The analysis was conducted at a constant temperature of 30 °C, with a flow rate set at 1 mL/min, and samples were injected using a 10 µL volume. The two enantiomer peaks (analyzed using a wavelength of 210 nm), as obtained with the synthesized reference, eluted at 23.8 min and 25.5 min. Based on the consumption of (*R*)-**1e**, the major enantiomer was attributed to (*R*)-2e at 25.5 min.

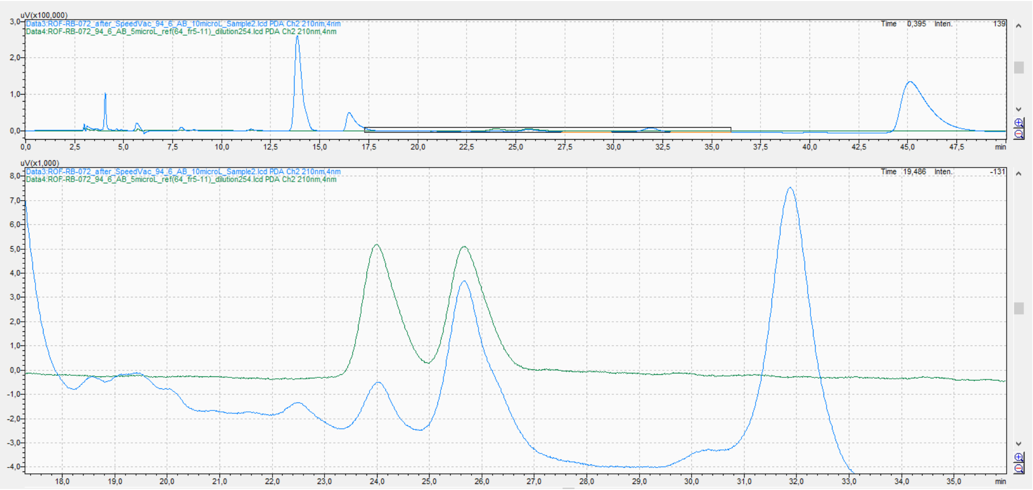

Figure S39. Overlay of trace obtained from derivatized *rac*-**2e** (green) and trace obtained from the biotransformation of *rac*-**1e** after derivatization (cyan) on chiral phase HPLC indicating formation of (*R*)-**2e** with 68% ee.

11. NMR

NMR measurements were performed on a Bruker Avance NEO 500 MHz NMR equipped with a Double Resonance Broadband Probe (BBI).

11.1. ^{31}P-NMR

Biotransformations to be analyzed by ^{31}P-NMR were prepared on 1 mL scale, analogously to the experiments for investigation of PpOplAB substrate scope. After 1 h, the reaction mixture was vortexed to denaturate the enzyme, which was pelleted by centrifugation (2 min). 25 µL of the supernatant was used for LC-MS analysis to determine conversion and samples for NMR analysis were prepared by mixing 630 µL of the supernatant with 70 µL D_2O.

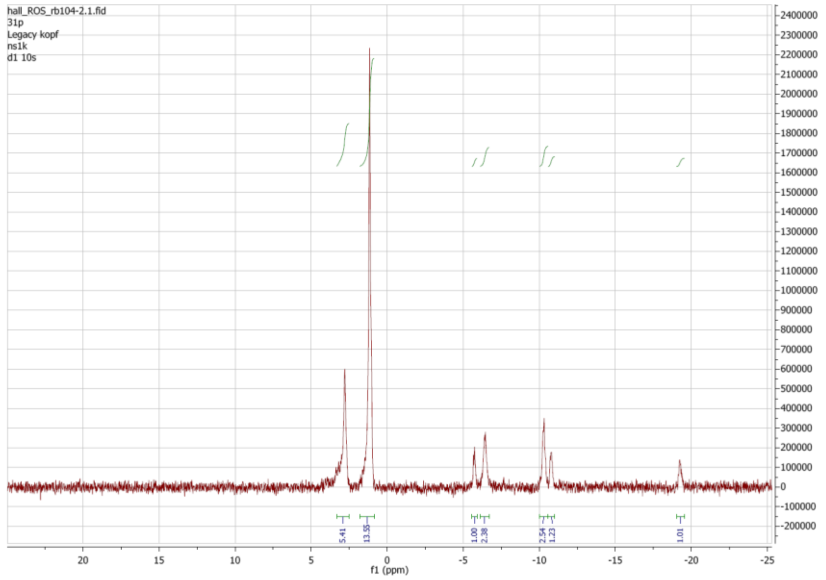

Figure S40. Spectrum from the biotransformation of 10 mM **1b** by PpOplAB in the presence of 12.5 mM ATP.

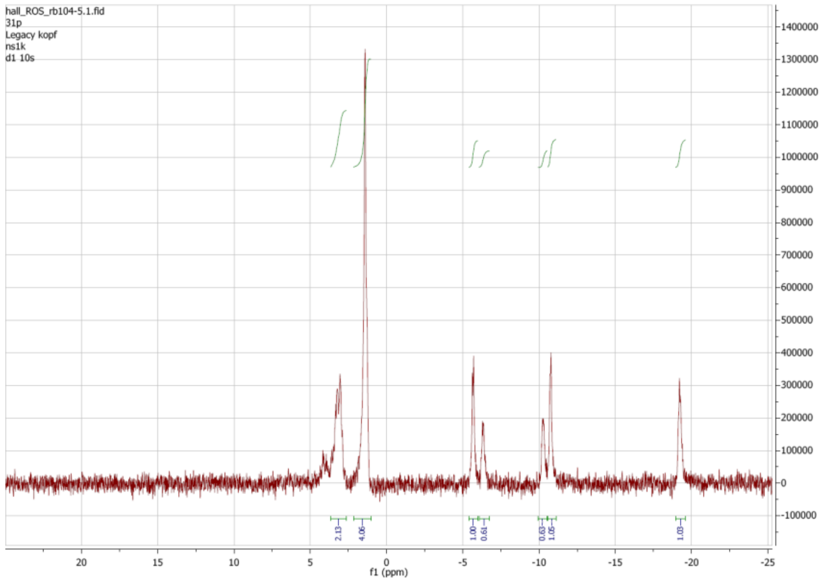

Figure S41. Spectrum from a sample of PpOplAB in the presence of 12.5 mM ATP without substrate.

11.2. ^{1}H-NMR

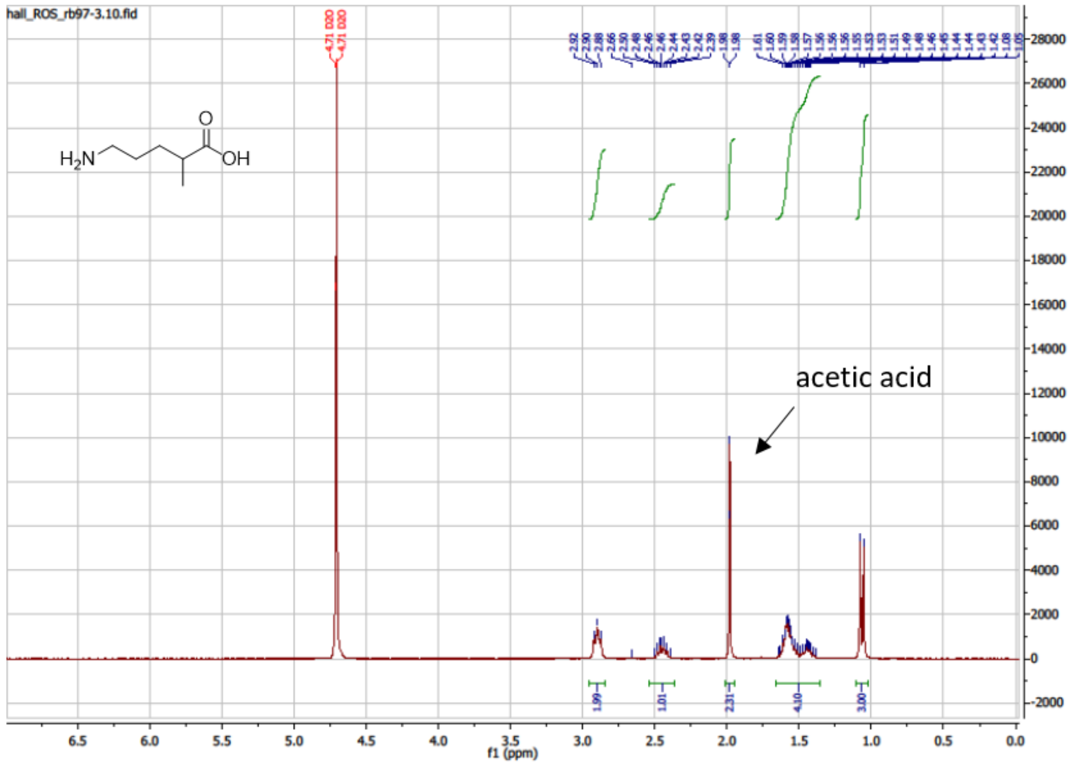

Figure S42. ^{1}H NMR spectrum of **2d** in D_2O with acetic acid as internal standard.

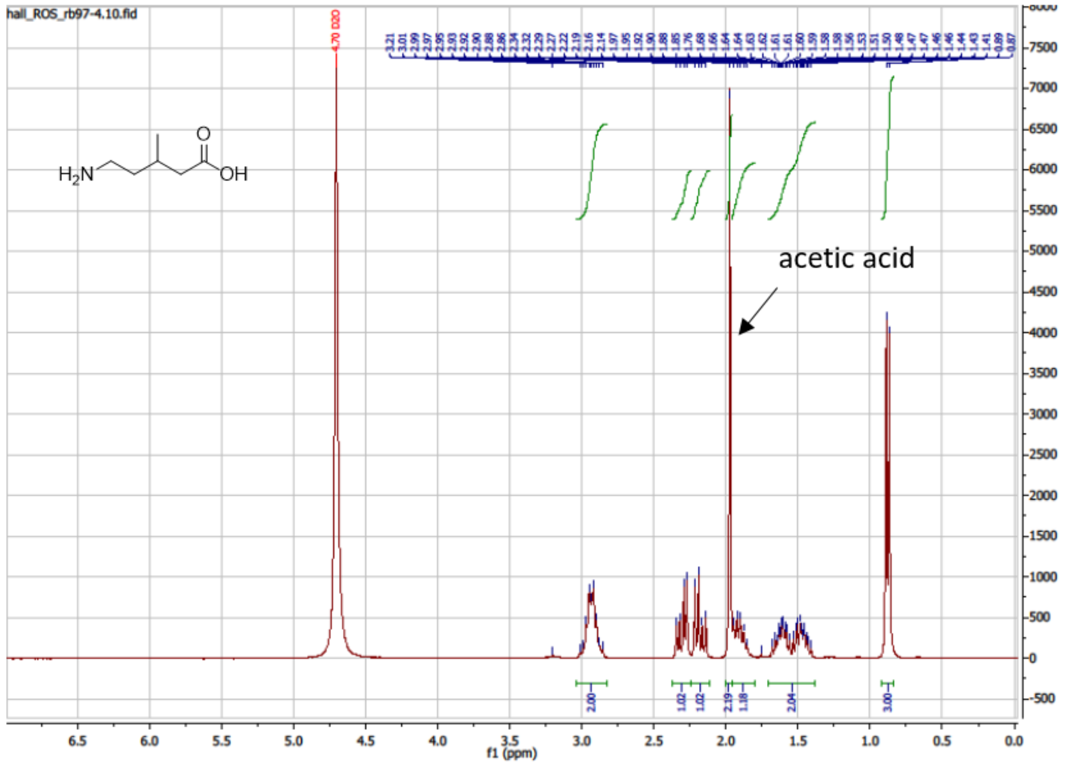

Figure S43. ^{1}H NMR spectrum of **2e** in D_2O with acetic acid as internal standard.

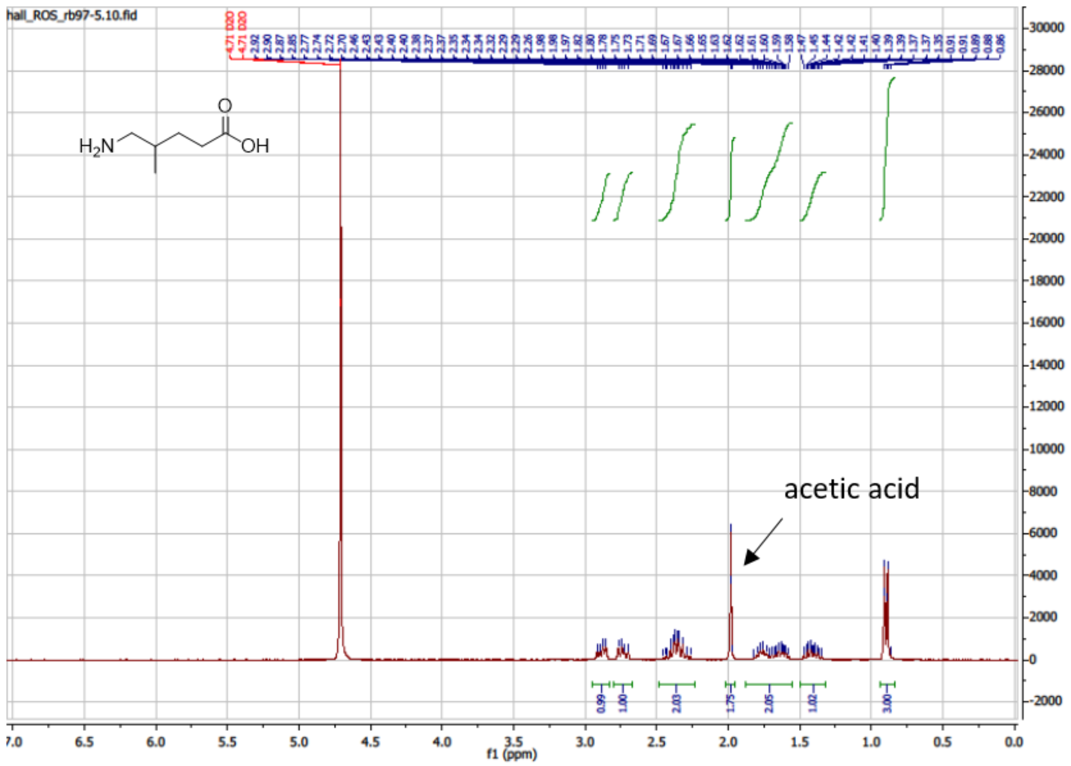

Figure S44. ^{1}H NMR spectrum of **2f** in D_2O with acetic acid as internal standard

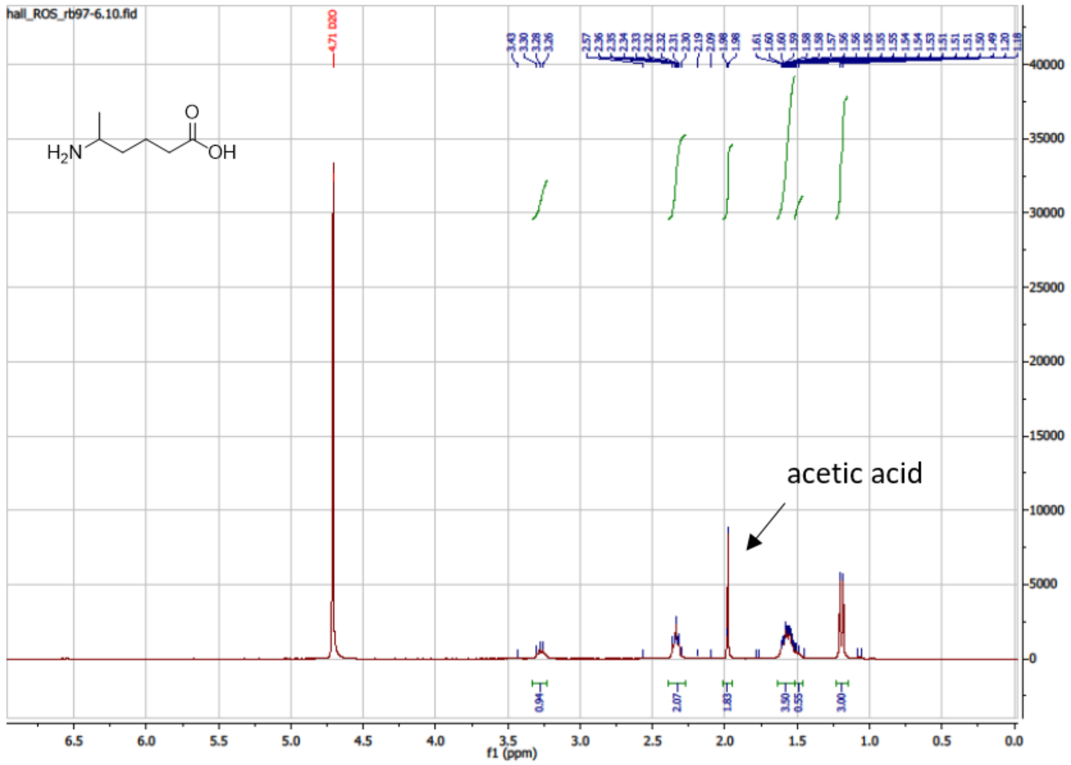

Figure S45. ^{1}H NMR spectrum of **2g** in D_2O with acetic acid as internal standard.

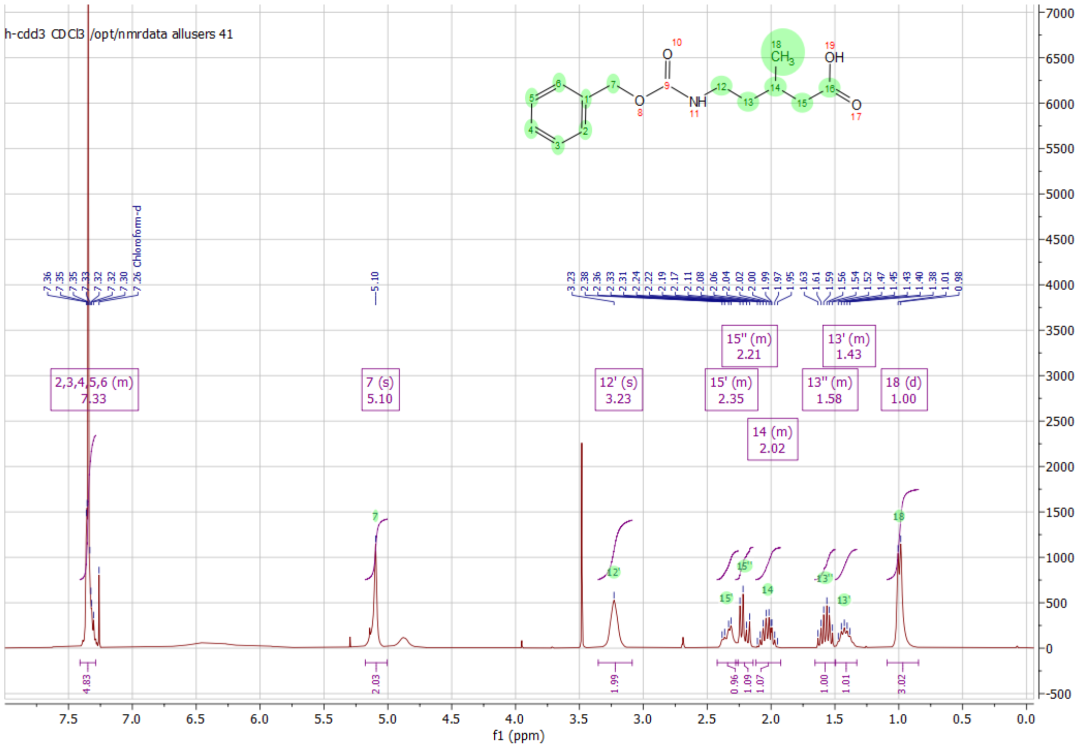

Figure S46. ^{1}H NMR spectrum of derivatized **2e** in $CDCl_3$.

12. Safety concerns

γ-Butyrolactam **1a** and δ-valerolactam **1b** are not innocuous compounds. Their safety datasheets indicate hazard pictograms GHS07 (exclamation mark: irritant, sensitising, harmful) and GHS08 (health hazard: long-term health hazard) for **1a** and GHS07 (exclamation mark: irritant, sensitising, harmful) for **1b**.

References

1 B. Nocek, S. Kochinyan, M. Proudfoot, G. Brown, E. Evcokimova, J. Osipiuk, A. M. Edwards, A. Savchenko, A. Joachimiak and A. F. Yakunin, *Proc. Natl. Acad. Sci. USA*, 2008, **105**, 17730.

2 M. I. Attia, C. Herdeis and H. Bräuner-Osborne, *Molecules*, 2013, **18**, 10266

3 R. Caputo, E. Cassano, L. Longobardo and G. Palumbo, *Tetrahedron*, 1995, **51**, 12337

4 T. Wagener, L. Lückemeier, C. G. Daniliuc and F. Glorius, *Angew. Chem. Int. Ed.*, 2021, **60**, 6425

5 M. Pickl, R. Marín-Valls, J. Joglar, J. Bujons and P. Clapés, *Adv. Synth. Catal.*, 2021, **363**, 2866
